# “Locking” CDK9 inhibition by activating integrator function in HIV transcription

**DOI:** 10.1016/j.isci.2026.117894

**Published:** 2026-10-17

**Authors:** Zichong Li, Francisco J. Zapatero-Belinchón, Patrik Geleziunas, Melanie Ott

**Affiliations:** 1 Gladstone Infectious Disease Institute, San Francisco, CA 94158, USA; 2 Department of Medicine, University of California, San Francisco, San Francisco, CA 94143, USA; 3 Biohub, San Francisco, CA 94158, USA; 4 University of Toronto, Toronto, ON, Canada

**Keywords:** HIV latency, block-and-lock, P-TEFb, CDK9 inhibitor, PP2A, integrator complex, AZD4573, SMAP-2

## Abstract

The positive transcription elongation factor b (P-TEFb) is a critical host factor for HIV transcription, yet pharmacological inhibition of its CDK9 subunit yields transient suppression with cytotoxicity. We identify that the integrator-PP2A complex enhances the potency and durability of CDK9 inhibition without added toxicity. Activation of PP2A with the small-molecule SMAP-2, combined with a low dose of the CDK9 inhibitor AZD4573, suppresses HIV transcription in CD4^+^ T cells, persisting for 7 days after drug withdrawal. Mechanistically, the drug combination increases integrator recruitment and repressive chromatin marks, decreases RNA polymerase II occupancy and C-terminal domain (CTD) phosphorylation at HIV promoter, and reduces short and long HIV transcripts, without global T cell activation, proliferation or broad transcriptome changes. The drug combination’s HIV-inhibiting effect is somewhat resistant against elevated HIV activity in the presence of cocaine or morphine. These findings reveal cooperative P-TEFb inhibition and integrator activation as a promising strategy for durable HIV silencing.

## Highlights

• AZD4573 and SMAP-2 synergistically suppress HIV transcription
• Combined treatment durably blocks HIV reactivation after drug withdrawal
• Integrator recruitment and repressive chromatin reinforce HIV silencing
• HIV suppression occurs without broad T cell activation or transcriptome changes

## Graphical abstract

**Figure:**
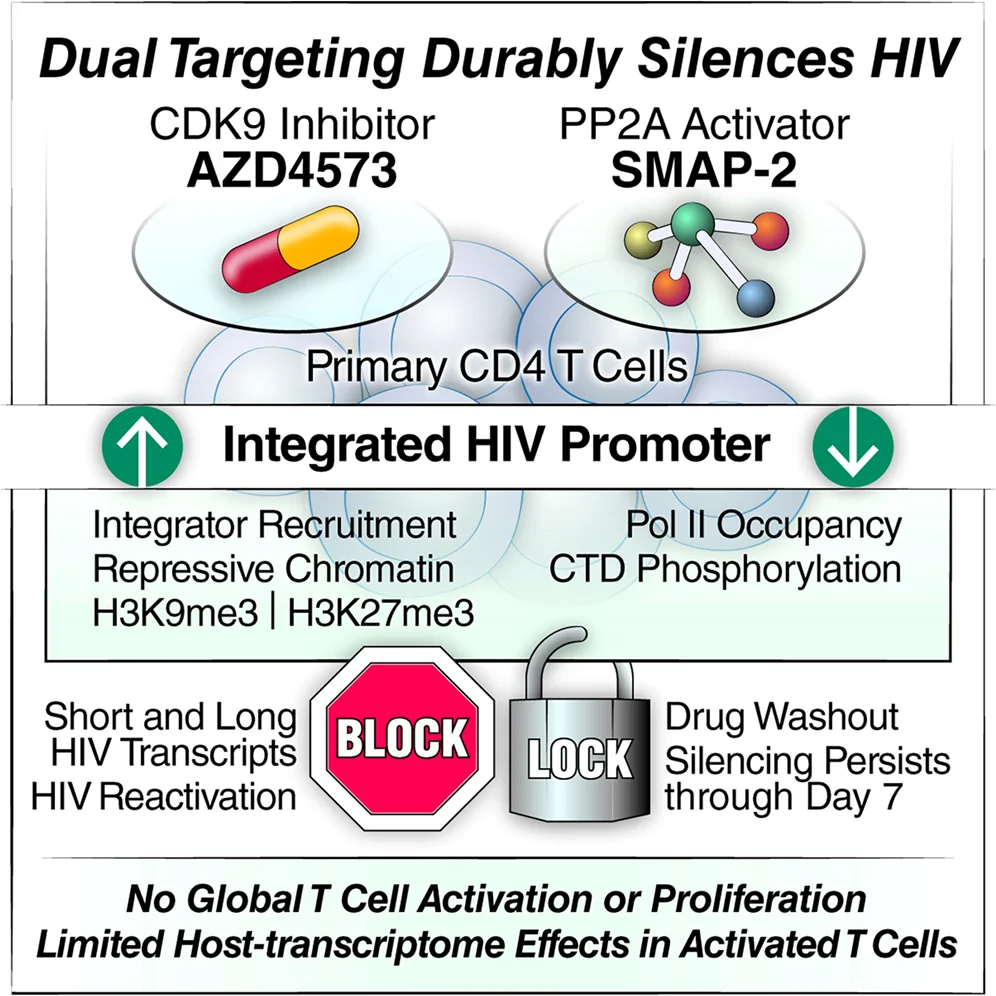

## Teaser

Microbiology; Cell biology

## Introduction

People living with HIV must adhere to lifelong antiretroviral therapy (ART) because a small population of reservoir cells persists and drives viral rebound upon treatment interruption.^1^ As current ART regimens do not include transcription inhibitors, HIV in reservoir cells remains transcriptionally active^2^^,^^3^ and contributes to chronic immune activation and rapid rebound.^4^

Genome-wide studies have identified numerous host factors that regulate HIV transcription.^5^^,^^6^^,^^7^^,^^8^^,^^9^^,^^10^ These findings have inspired cure strategies such as “shock-and-kill” that reactivates latent HIV for elimination, and “block-and-lock” that enforces deep silencing. Studies of elite controllers show that viral integration into heterochromatin with repressive marks enables sustained, drug-free control.^11^ This demonstrates that strong transcriptional repression can achieve a functional cure.^12^^,^^13^^,^^14^^,^^15^^,^^16^ HIV transcription is governed by cooperation between virally encoded protein and RNA and host transcriptional machinery.^4^ Recruitment of RNA polymerase II (Pol II) to the integrated proviral promoter can be blocked by nucleosome remodeling complexes.^17^ Even after initiation, Pol II typically pauses at nucleotide +59, where the nascent viral RNA forms the *trans*-activation response (TAR) stem-loop. Productive elongation requires the viral protein Tat, which binds TAR and recruits the positive transcription elongation factor b (P-TEFb), a CDK9-cyclin T1 kinase complex. P-TEFb is indispensable for Pol II elongation and the production of full-length HIV RNA. This central role makes CDK9 inhibition a powerful way to block HIV transcription.^18^ Accordingly, perturbations that disrupt P-TEFb activity or its functional engagement by Tat can profoundly suppress HIV transcriptional output.^19^^,^^20^ However, as P-TEFb is similarly essential for normal gene expression, global CDK9 inhibition results in significant toxicity.^21^ Thus, strategies that modulate P-TEFb activity in a more selective way, or combine lower doses of CDK9 inhibitors with drugs targeting cooperating factors, are critical for making transcriptional repression of HIV clinically viable.

Recent studies in cancer cells have shown that pharmacological activation of the protein phosphatase PP2A synergizes with CDK9 inhibition to enhance transcriptional repression.^22^ This observation prompted us to test whether the same drug combination could induce durable silencing of HIV. In support of this idea, our own recent screens^7^ had identified host elongation factors and subunits of the PP2A-Inregrator complex synergistically regulate HIV transcription. Here, we show that pharmacological activation of the PP2A-integrator axis with the PP2A agonist SMAP-2, in combination with the CDK9 inhibitor AZD4573, enforces durable repression of HIV transcription in primary CD4^+^ T cell latency models. This suppressive effect persists after drug withdrawal and extends to cells exposed to cocaine or morphine, drugs that normally trigger HIV reactivation. These findings extend the PP2A-integrator/CDK9 framework from cancer transcriptional regulation to HIV block-and-lock strategies and support dual targeting of these pathways as an approach to durable HIV silencing.

## Results

### Synergistic regulation of HIV transcription by elongation factors and integrator

Our CRISPRi screening previously identified integrator and elongation factors FTSJ3 and PSMD3 as regulators of HIV latency.^7^^,^^9^ To validate these interactions, we knocked down *INTS2* (encoding a subunit of integrator), *FTSJ3*, and *PSMD3* in J-Lat A2 ^23^ cells via RNAi. Depletion of each target was confirmed by western blot (Figure S1). *INTS2* depletion alone modestly reversed latency, but co-depletion with either *FTSJ3* or *PSMD3* produced synergy well beyond additive expectations, as defined by the Bliss independence model (Figures 1A–1C and 1E). In contrast, knockdown of *FTSJ3* and *PSMD3* together produced only an additive effect (Figures 1D and 1E). Thus, elongation and termination factors act cooperatively to maintain HIV latency. Importantly, knockdown of *FTSJ3* or *PSMD3* did not alter the protein levels of key transcriptional regulators including CDK9 and cyclin T1, nor did it affect INTS2 levels (Figure S1). These controls exclude the possibility of indirect effects on P-TEFb or integrator abundance.

**Figure 1:**
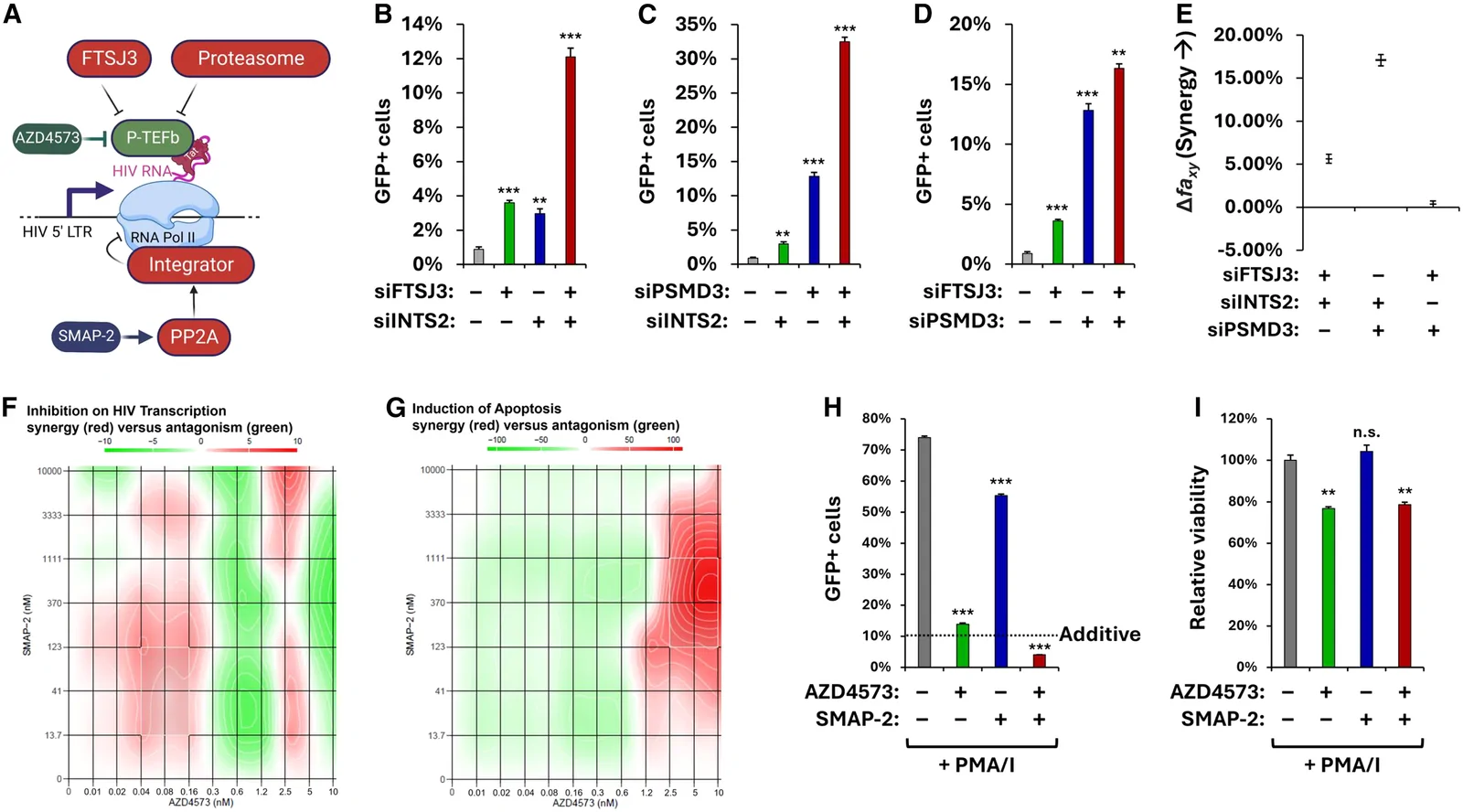
Synergistic interactions between integrator and regulators of P-TEFb control the reactivation or silencing of the latent HIV LTR (A) Schematic of P-TEFb (CDK9 + cyclin T1) and Integrator pathways in HIV transcription and their modulation by study drugs. (B–D) J-Lat A2 cells harboring GFP under the control of a latent HIV LTR were nucleofected with the indicated siRNAs and subjected to flow cytometry to measure the percentage of GFP^+^ cells. (E) Bliss independence test for data in B–D: Δfa_xy_ values were calculated using the Bliss independence model, where the predicted additive effect (fa_xy,p_) of the pairwise combination of two drugs (each individual drug’s effect is fa_x_ or fa_y_) was defined as fa_xy,p_ = fa_x_ + fa_y_ − (fa_x_ × fa_y_), and synergy was calculated as Δfa_xy_ = fa_xy,o_ − fa_xy,p_ (in which fa_xy,o_ represents the observed combinatorial effect). Positive Δfa_xy_ values indicate synergy beyond additivity. (F and G) J-Lat A2 cells were pre-treated with a matrix of CDK9 inhibitor AZD4573 (0–10 nM) and PP2A stimulator SMAP-2 (0–10 μM) for 2 h, followed by stimulation with 81 nM PMA and 0.1 μM ionomycin in the presence of the drugs. GFP expression (F) and annexin V staining (G) were monitored every hour for 16 h using the Incucyte system. Heatmaps display ZIP synergy scores, with red indicating synergy and green indicating antagonism. Multiple concentration pairs exhibited strong synergy in suppressing HIV reactivation, while no corresponding synergy was observed in apoptosis, suggesting the effect is not due to cytotoxicity. (H and I) J-Lat A2 cells were treated with 5 nM AZD4573, 5 μM SMAP-2, their combination, or DMSO for 2 h and then 25 nM PMA + 0.5 μM ionomycin were added for 1 day. The cells were then subjected to flow cytometry to quantify GFP^+^ cells and relative viability (normalized to the DMSO group). The dotted line in (H) marks the predicted effect of the drug combination if the drugs’ effects were additive, based on the Bliss independence model. In (B)–(E), (H) and (I) data are represented as mean ± SD from three technical replicates, asterisks denote levels of statistical significance compared with the DMSO groups calculated by two-tailed Student’s *t* test (∗: *p* < 0.05, ∗∗: *p* < 0.01, and ∗∗∗: *p* < 0.001). See also Figures S1, S2, S3, S4, and S5; Table S1.

To assess how INTS2 depletion affects integrator complex assembly, we performed co-immunoprecipitation of INTS11 followed by detection of associated subunits. INTS2 knockdown reduced the association of INTS6 and INTS8 with INTS11, whereas the association of INTS4 with INTS11 was retained (Figure S2). These findings indicate that INTS2 depletion selectively perturbs interactions among integrator subunits, including components of the PP2A-associated module. However, this experiment does not determine whether the INTS11 endonuclease module remains functionally competent or whether integrator-mediated nascent RNA cleavage is preserved.

We next tested whether the synergy between integrator and elongation factors could be reproduced pharmacologically. For this, we used AZD4573,^23^ a selective CDK9 inhibitor, and SMAP-2,^22^^,^^24^^,^^25^ a PP2A agonist that activates integrator (Figure 1A). Dose-response analysis of individual compounds confirmed that AZD4573 suppressed reactivation at 5 nM (Figures S3A, S3B, S3D, and S3E) but was cytotoxic at 10 nM (Figures S3C and S3F), while SMAP-2 had little activity on its own and was cytotoxic at 10 μM (Figures S3G and S3H).

High-throughput titration across a concentration matrix revealed several dose pairs with strong synergy in suppressing PMA/I-induced HIV reactivation (red peaks in Figure 1F; Table S1). The multimodal distribution of the synergy scores across the matrix likely reflects a balance between complementary transcriptional inhibition and emerging cytotoxicity at higher concentrations that confines optimal synergy to a defined window. Within this window, the most robust synergy without corresponding increases in annexin V staining (Figure 1G) is 3 nM AZD4573 and 3 μM SMAP-2. A parallel flow cytometry-based matrix independently confirmed this pattern, supporting that the dual treatment enhances HIV silencing without driving cytotoxicity (Figure S4).

Although peak synergy occurred at 3 nM AZD4573 and 3 μM SMAP-2 in the high-throughput titration screen, we selected 5 nM AZD4573 and 5 μM SMAP-2 for subsequent experiments. This adjustment improved reproducibility and tolerability under low throughput conditions, and minimized potential differences due to assay formats and culture setups. Testing these selected doses under PMA/I stimulation showed that AZD4573 or SMAP-2 alone reduced reactivation from ∼74% to 14% and 55%, respectively, whereas the combination drove reactivation down to 4%, well below the Bliss-predicted additive effect of 10.4% (Figure 1H). Importantly, this potent synergy occurred without added cytotoxicity (Figure 1I), demonstrating that dual targeting of CDK9 and integrator provides a highly specific block to HIV reactivation.

Overall, these findings are consistent with our previous CRISPRi results,^7^ which identified synergistic interactions between elongation and termination factors in regulating HIV latency. The present results extend this observation by validating the synergy using RNAi and establishing the rationale for pharmacologically co-targeting these pathways. Of note, SMAP-2 also synergized with SNS-032, a multi-CDK inhibitor with activity against CDK2, CDK7, and CDK9, to reduce HIV reactivation (Figure S5). Because SNS-032 is not selective for CDK9,^26^^,^^27^ this result provides an additional example of pharmacological synergy with SMAP-2 but does not establish that the interaction is specifically attributable to CDK9 inhibition.

### AZD4573 and SMAP-2 inhibit different steps of HIV transcription

P-TEFb promotes transcription elongation,^28^ while integrator induces premature termination.^29^^,^^30^ These orthogonal functions suggest that CDK9 inhibition and PP2A activation might inhibit different steps in HIV transcription. To test this, we used RT-qPCR to separately quantify short initiated (∼59 nt) and long elongated (≥ 190 nt) HIV transcripts in PMA/I-treated J-Lat A2 cells (Figure 2A).

**Figure 2:**
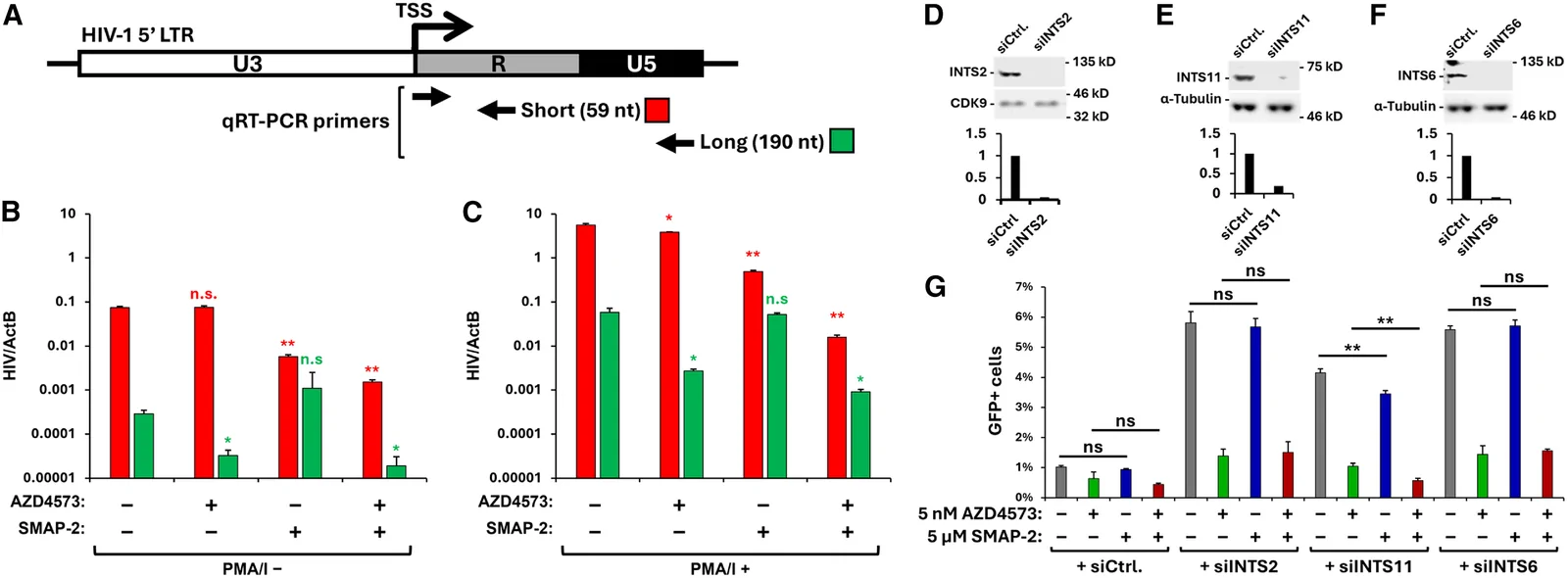
Cooperative inhibition of HIV transcription by SMAP-2 and AZD4573 (A) A schematic diagram showing the HIV-1 5′ LTR, the transcription start site (TSS) and the location of the primer sets that were used in RT-qPCRs to quantify the short (59 nt) and long (190 nt) HIV-1 transcripts. (B and C) RT-qPCR quantification of the short (red bars) and long (green bars) HIV-1 transcripts from J-Lat A2 cells using the specific primers shown in (A). The qPCR signals were normalized to that of *ActB*. Each column represents the average of three replicate RT-qPCR reactions, and the error bars indicate means ± standard deviation (SD). J-Lat A2 cells were nucleofected with the indicated siRNAs, treated with the indicated drugs 1 day after nucleofection, and subjected to flow cytometry to quantify GFP^+^ cells the next day (G). An aliquot of the nucleofected cells was subjected to western blot to monitor the expression levels of the indicated proteins 2 days after nucleofection, and densitometry conducted to quantify the knockdown efficiency of the targeted proteins normalized to the loading controls (D–F). In (B), (C), and (G) data are represented as mean ± SD from three technical replicates, asterisks denote levels of statistical significance calculated by two-tailed Student’s *t* test (∗: *p* < 0.05, ∗∗: *p* < 0.01, and ∗∗∗: *p* < 0.001).

As shown in Figure 2B (spontaneous HIV transcription without reactivation) and 2C (PMA/I-stimulated HIV transcription), in J-Lat A2 cells, AZD4573 selectively reduced long transcripts without affecting short RNAs, consistent with its inhibition of elongation; SMAP-2 reduced short HIV transcripts, indicating an overall decrease in early transcriptional output; the combination of AZD4573 and SMAP-2 suppressed both transcript classes (Figures 2B and 2C).

Given the known roles of integrator and PP2A in promoter-proximal pausing and premature termination,^31^ the RT-qPCR results above likely reflect enhanced termination and/or reduced productive engagement of RNA polymerase II at the HIV LTR, rather than a strictly initiation-specific effect. Because Tat-dependent HIV transactivation critically relies on P-TEFb recruitment to TAR for productive elongation, these findings also suggest that the presence of a CDK9 inhibitor in the drug combination functionally antagonizes Tat-driven transcriptional activation, a hypothesis we address further down.

To dissect the contributions of distinct integrator modules, we knocked down *INTS2* (scaffolding), *INTS6* (PP2A adaptor), and *INTS11* (endonuclease) individually with siRNAs (Figures 2D–2F). Depletion of any subunit increased HIV transcription (Figure 2G gray bars), confirming the role of integrator in maintaining latency.^9^^,^^31^ However, SMAP-2 failed to suppress HIV transcription following depletion of INTS2 or the PP2A adaptor INTS6, whereas its suppressive activity was retained following INTS11 depletion (Figure 2G). These findings support a greater requirement for the structural and PP2A-associated modules of integrator than for INTS11 in SMAP-2-mediated repression under the knockdown conditions tested. Because INTS11 depletion may have been incomplete and nascent RNA cleavage was not measured directly, these experiments do not establish that integrator endonuclease activity is dispensable.

### Dual targeting of P-TEFb and PP2A cooperatively “blocks-and-locks” HIV reactivation in primary CD4^+^ T cells

We next tested whether the HIV-silencing effects of AZD4573 and SMAP-2 extend to a primary CD4^+^ T cell model of latency^32^ (Figure 3A). In this system, latency is reversed by CD3/CD28 antibody stimulation, which globally activates T cells and provides a stronger stimulus than PMA/I.

**Figure 3:**
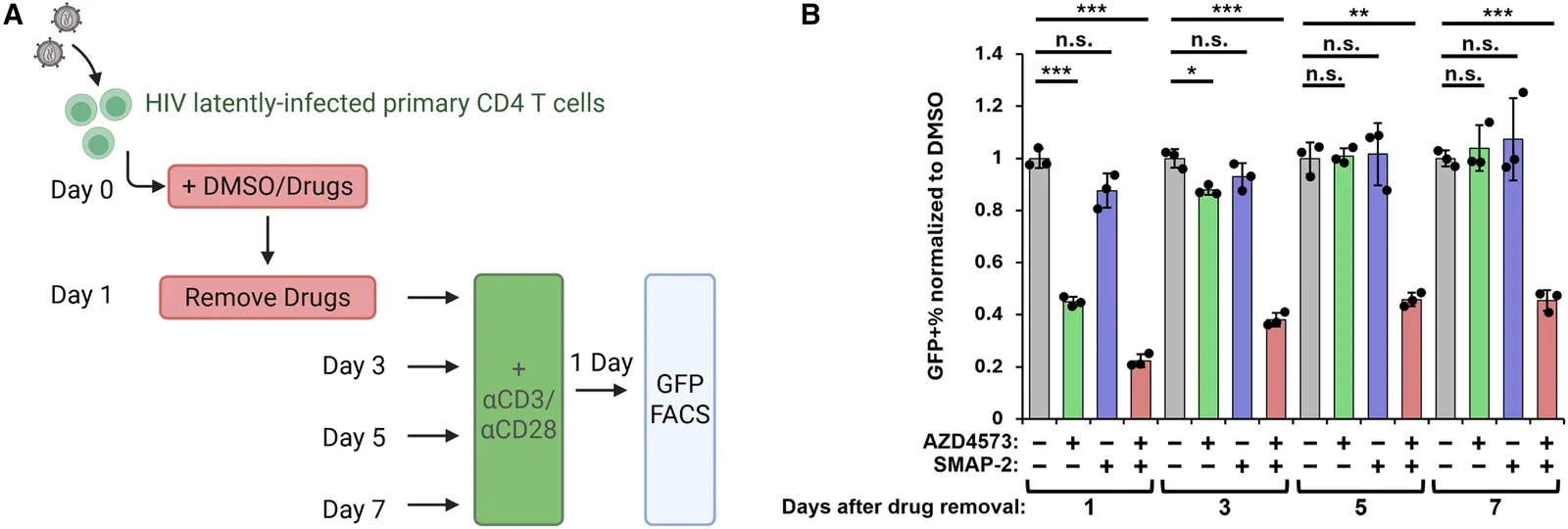
AZD4573 and SMAP-2 cooperatively block-and-lock the reactivation of latent HIV in primary CD4^+^ T cells (A) Schematic summary: primary resting CD4^+^ T cells latently infected with a single-round HIV-GFP reporter virus were treated with 0.1% DMSO (as control), 5 nM AZD4573, 5 μM SMAP-2 or their combination. On the next day, the cells were washed and cultured in drug-free medium. On the same day and 3, 5, and 7 days after, we took an aliquot of the cells and stimulated their HIV expression through CD3/CD28 antibody-induced T cell activation for one day and then harvested the cells to quantify HIV-expressing cells with GFP flow cytometry. (B) The fraction of GFP^+^ cells in each group was quantified with flow cytometry and normalized to the DMSO group. Data are represented as mean ± SD from three biological replicates (CD4^+^ T cells from *n* = 3 donors). In (B) asterisks denote levels of statistical significance compared with the DMSO groups calculated by two-tailed Student’s *t* test (∗: *p* < 0.05, ∗∗: *p* < 0.01, and ∗∗∗: *p* < 0.001).

Pretreatment for 1 day with 5 nM AZD4573 + 5 μM SMAP-2 suppressed antibody-induced HIV reactivation by ∼80% (Figure 3B, day 1). Notably, this effect persisted: even after 6 additional days in drug-free medium, reactivation remained ∼50% lower than in DMSO-treated controls (Figure 3B, day 7). By contrast, pretreatment with AZD4573 alone reduced reactivation initially but the effect disappeared within 3 days of drug withdrawal (Figure 3B, days 3, 5, and 7).

These results show that the combination of AZD4573 and SMAP-2 not only blocks HIV reactivation in primary CD4^+^ T cells but also maintains this blocking effect after drug removal, providing evidence for a cooperative “block-and-lock” effect.

### AZD4573 and SMAP-2 remodel histone modifications on HIV LTR

To investigate why the HIV-silencing effect of AZD4573 and SMAP-2 persists for up to 7 days after drug withdrawal (Figure 3B), we performed ChIP on latently infected primary CD4^+^ T cells, treating them as in Figure 3A and harvesting on days 1 and 7. SMAP-2 transiently increased recruitment of the integrator scaffolding subunit INTS11 (Figure 4A). Crucially, integrator occupancy was only maintained through day 7 when SMAP-2 was combined with AZD4573 (Figure 4A). This sustained INTS11 binding by the combination therapy correlated with marked, prolonged reductions in occupancy by total Pol II (Figure 4B), as well as its Ser5- (Figure 4C) and Ser2-phosphorylated forms (Figure 4D). These effects were antibody-specific, as ChIP assays with a non-immunized IgG failed to pull down the LTR (Figure 4E).

**Figure 4:**
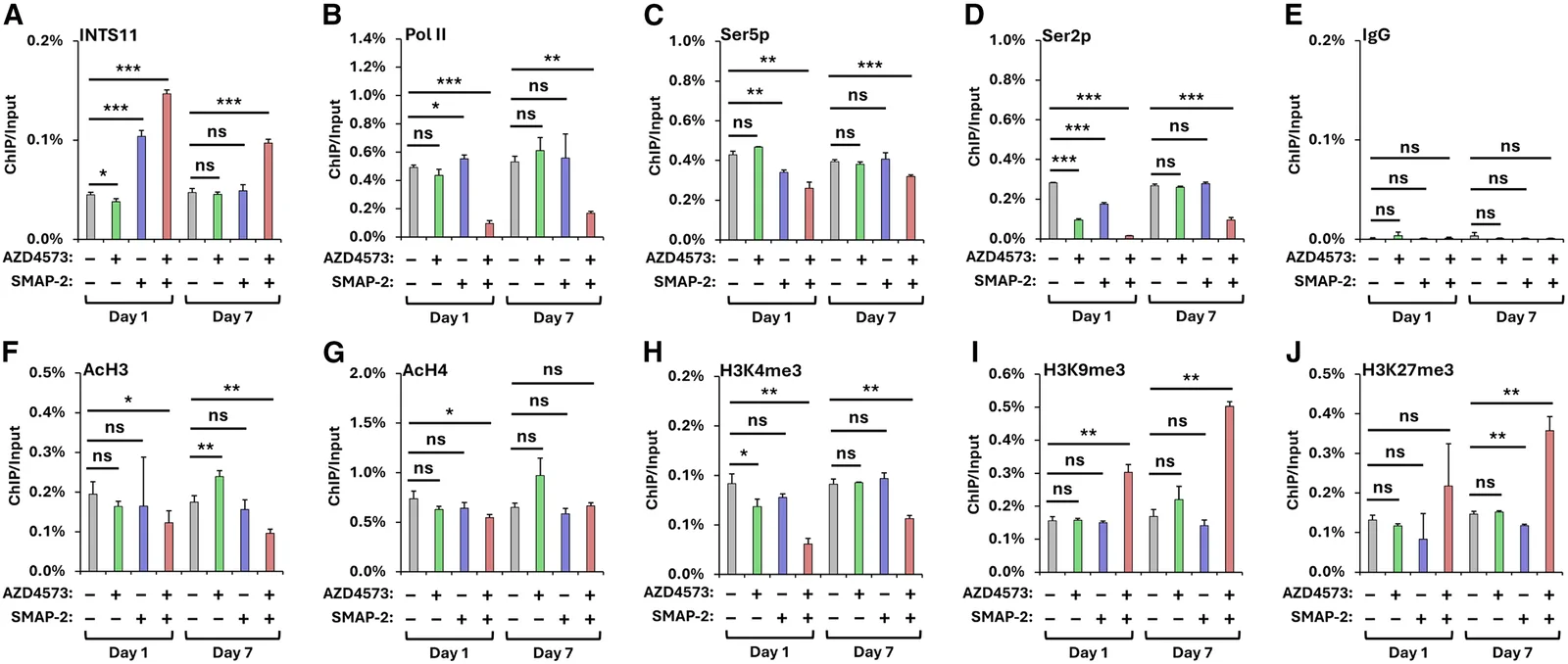
Epigenetic changes induced by the combination of AZD4573 and SMAP-2 on the HIV LTR Primary CD4^+^ T cells latently infected with HIV were treated with either 5 nM AZD4573 or 5 μM SMAP-2 alone or in combination for 1 day, harvested on days 1 and 7 and subjected to ChIP-qPCR using normal rabbit immunoglobulin G (IgG) (for control) or antibodies that specifically recognize the indicated proteins to quantify their occupancy of the Nuc-1 region of HIV LTR. The ChIP-qPCR signals were normalized to input DNA. In (A–J) data are represented as mean ± SD from three technical replicates, asterisks denote levels of statistical significance compared with the DMSO groups calculated by two-tailed Student’s *t* test (∗: *p* < 0.05, ∗∗: *p* < 0.01, and ∗∗∗: *p* < 0.001).

Concurrently, the drug combination reduced activating histone modifications, specifically AcH3 (Figure 4F) and H3K4me3 (Figure 4H) across both time points, and AcH4 on day 1 (Figure 4G), supporting a shift toward a repressive chromatin state. Repressive marks progressively accumulated in parallel, with H3K9me3 increasing on days 1 and 7 (Figure 4I) and H3K27me3 on day 7 (Figure 4J). The apparent coexistence of H3K9me3 and H3K27me3 likely reflects population-level averaging across cells, where distinct repressive mechanisms may operate in different subsets rather than co-occupying the same chromatin region simultaneously.

Together, these findings demonstrate that AZD4573 and SMAP-2 establish a stable, integrator-dependent repressive chromatin state, characterized by decreased Pol II recruitment, reduced histone acetylation, and increased repressive histone methylation, to sustain the suppression of HIV transcription.

### AZD4573 and SMAP-2 suppress HIV without inducing T cell activation, proliferation, or broad host transcriptional changes

Because AZD4573 and SMAP-2 target essential cellular proteins, we assessed whether their combined use causes cytotoxicity or global T cell activation under the same conditions as in latency-reversal assays (Figure 3A). After 24 h of treatment with 5 nM AZD4573 and 5 μM SMAP-2, the drug combination did not induce CD25 and only marginally induced CD69 expression on primary CD4^+^ T cells (Figure 5A, middle), in sharp contrast to the strong activation caused by anti-CD3/CD28 stimulation (Figure 5A, right).

**Figure 5:**
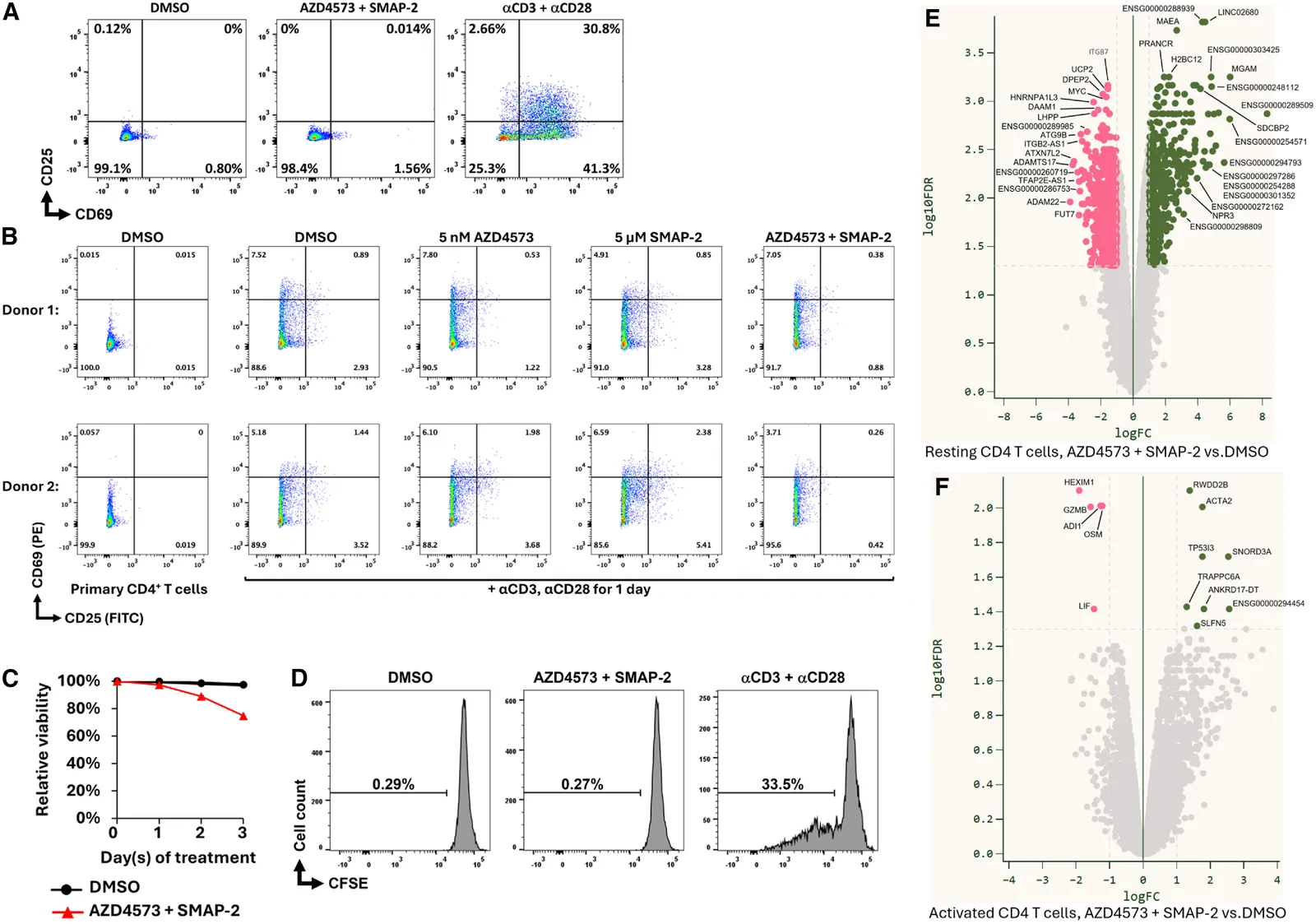
Effects of the combination of AZD4573 and SMAP-2 on primary CD4^+^ T cell activation, proliferation and viability (A) Primary CD4^+^ T cells (from 1 donor) were treated with DMSO, 5 nM AZD4573 + 5 μM SMAP-2, or antibodies targeting CD3 and CD28 for 24 hr. The cell surface expression of the T cell activation markers CD69 and CD25 was examined by immunostaining and flow cytometry. (B) Primary CD4^+^ T cells (*n* = 2 donors) were treated with DMSO, 5 nM AZD4573, 5 μM SMAP-2, or their combination together with antibodies targeting CD3 and CD28 for 24 hr. The cell surface expression of the T cell activation markers CD69 and CD25 was examined by immunostaining and flow cytometry. (C) Primary CD4^+^ T cells (from 1 donor) were treated with DMSO or 5 nM AZD4573 + 5 μM SMAP-2 for 3 days. An aliquot of cells from each treatment was collected on the indicated days, stained with a LIVE/DEAD dye, and subjected to flow cytometry to quantify the percentage of live cells. (D) Primary CD4^+^ T cells (*n* = 1 donor) were stained with CFSE, treated with either DMSO, 5 nM AZD4573 + 5 μM SMAP-2, or antibodies targeting CD3 and CD28 for 24 hr, and then cultured under drug-free conditions for 3 additional days and analyzed by flow cytometry. (E and F) Primary CD4^+^ T cells from three HIV-negative donors (*n* = 3) were analyzed by RNA-seq following drug treatment. Cells were either maintained in a resting state (E) or activated for 2 days with anti-CD3/CD28 antibodies (F), then treated for 24 h with 0.1% DMSO or 5 nM AZD4573 + 5 μM SMAP-2. Differentially expressed genes consistently identified across all three donors are shown as volcano plots. See also Figures S6 and S7; Table S2.

To directly test whether the compounds affect T cell activation induced by TCR-stimulation, AZD4573 and SMAP-2 were added together with anti-CD3/CD28 antibodies. Under these conditions, the combination significantly reduced induction of CD25 and CD69 compared to antibody stimulation alone (Figures 5B and S6), consistent with prior reports that CDK9 inhibition suppresses T cell activation.^33^

The combination was well tolerated during the initial 24-h treatment but cell death increased beginning on day 2 of treatment (Figure 5C). Carboxyfluorescein succinimidyl ester (CFSE) staining showed no evidence of proliferation following drug exposure and an additional 4 days in drug-free medium (Figure 5D).

Together, these results demonstrate that AZD4573 and SMAP-2 cooperatively suppress HIV reactivation without inducing global T cell activation or proliferation and are tolerated by primary CD4^+^ T cells during short-term exposure.

To further assess potential effects of the drug combination on host pathways, we performed transcriptome profiling of resting and activated CD4^+^ T cells treated with the combination of AZD4573 and SMAP-2 versus DMSO. In resting CD4^+^ T cells (Figure 5E), numerous transcripts were differentially expressed upon treatment (Table S2), the majority corresponding to unannotated protein-coding loci or long non-coding RNAs. This pattern is consistent with the known functions of the integrator complex, which regulates transcription and 3′-end processing of snRNAs and other non-coding RNA species.^34^ Among annotated genes, mRNA of *MYC*, a canonical P-TEFb target, was significantly reduced. In contrast, activated CD4^+^ T cells exhibited far fewer transcriptional changes, with *HEXIM1*, a bona fide P-TEFb-responsive gene, among the few downregulated by drug treatment (Figure 5F). Interestingly, transcription of the transcriptional co-repressor SLFN5 was upregulated in activated CD4^+^ T cells exposed to AZD4573 and SMAP-2. We validated *SLFN5* upregulation by RT-qPCR (Figure S7A), and *HEXIM1* downregulation by western blot (Figure S7B). Given *SLFN5*’s reported role in repressing HIV transcription through histone methylation,^35^ its induction by the drug combination may contribute to the reinforced block-and-lock phenotype.

Overall, these results indicate that while the drug combination can modulate select host genes, its global transcriptional impact in activated T cells remains limited under the conditions tested. Notably, the larger transcriptional response in resting T cells likely reflects their heightened sensitivity to perturbations in basal transcriptional machinery, consistent with their quiescent state and dependence on precise RNA-processing pathways.

### HIV-inhibiting effects of AZD4573 and SMAP-2 in the presence of cocaine and morphine

Substance use is common among people living with HIV and can enhance viral transcription, complicating latency control.^36^^,^^37^ To test whether AZD4573 and SMAP-2 remain effective under these conditions, we obtained primary CD4^+^ T cells from four different donors and infected them latently with HIV-GFP, before exposing them to AZD4573 and SMAP-2 in the presence or absence of cocaine or morphine (Figure 6A). We first confirmed that in the absence of cocaine or morphine, the combination of AZD4573 and SMAP-2 strongly suppressed PMA/I-induced HIV-GFP expression (Figure 6B), and this suppression persisted for at least one week after drug withdrawal (Figure 6C). Interestingly, AZD4573 alone did not significantly reduce PMA/I-induced HIV-GFP expression in these experiments (Figure 6B, AZD4573 group). SMAP-2 alone induced a more variable response across donors than did other conditions. This variation was driven by a single donor with elevated reactivation levels (Figure 6B, SMAP-2 group). However, across donors, SMAP-2 did not significantly differ from PMA/I alone (*p* = 0.33), indicating no consistent latency-reversing activity. This variability may reflect donor-specific differences in transcriptional responsiveness.

**Figure 6:**
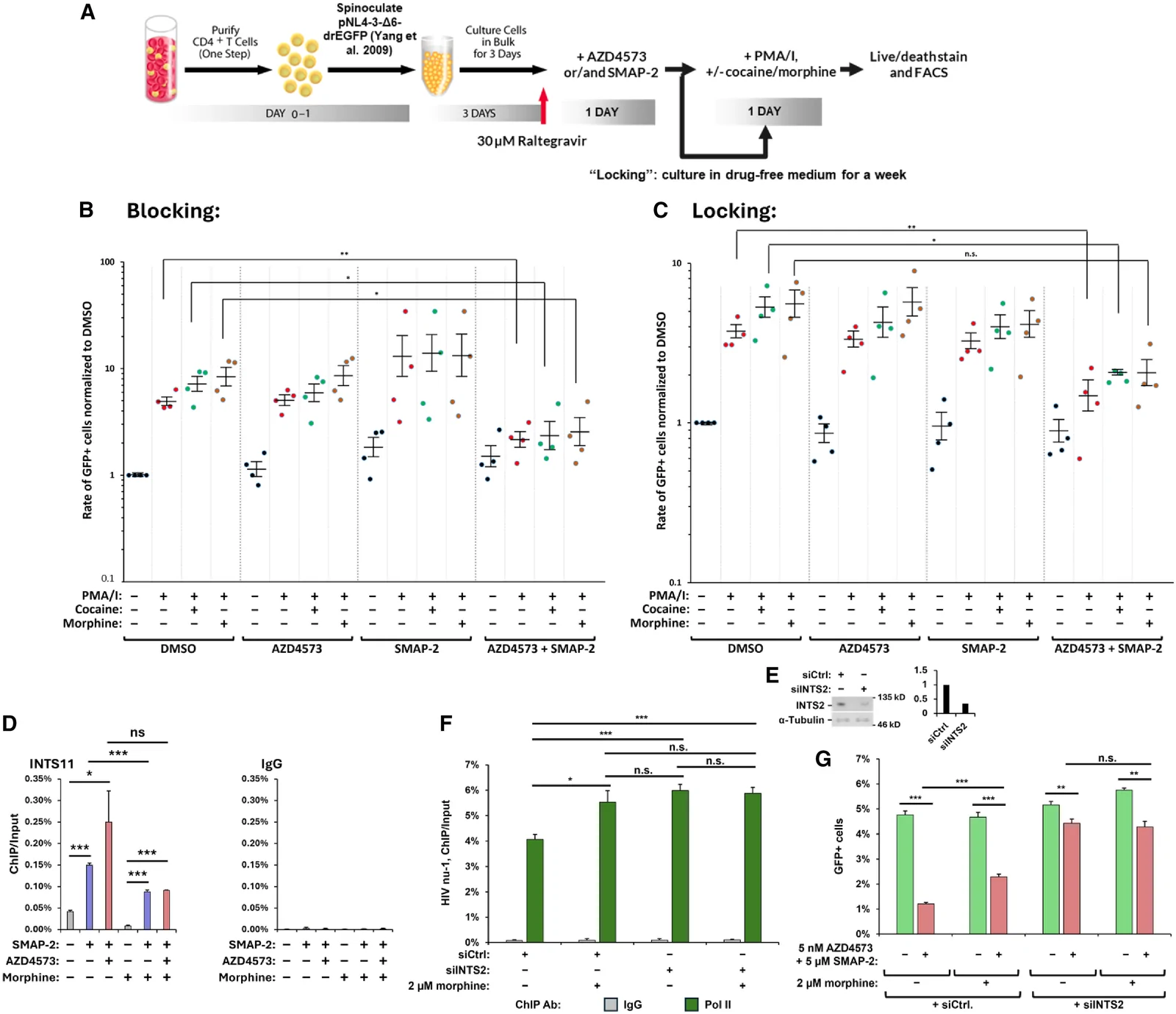
HIV-blocking effects of AZD4573 and SMAP-2 in the presence of cocaine and morphine (A) Schematic summary: primary resting CD4^+^ T cells latently infected with a single-round HIV-GFP reporter virus were treated with either 5 nM AZD4573 or 5 μM SMAP-2 alone or in combination. On the next day, for half of the cells, we induced HIV expression through treatment with 25 nM PMA + 0.5 μM ionomycin (PMA/I) for 1 day and then harvested the cells to quantify HIV transcription by GFP FACS. For the other half of the cells, we washed away the drugs, then maintained the cells in drug-free medium for 6 days, and finally did the same PMA/I treatment and GFP FACS to see if there is a lingering effect of the drug treatment. (B and C) HIV-1 transcription, as measured by the percentage of GFP^+^ cells, in indicated treatment groups. For each image, data were normalized to DMSO-only condition. Data are represented as mean of *n* = 4 donors ± standard error of the mean (to accommodate the usage of log value on the *y* axis). Significance was tested by two-tailed Student’s *t* test. ∗: *p* < 0.05, ∗∗: *p* < 0.01, ns, not significant. (D) An aliquot of the latently infected primary resting CD4^+^ T cells (from one donor) treated with the indicated drugs for one day was subjected to INTS11 or IgG chromatin immunoprecipitation (ChIP) assays. (E–G) Viral infection was performed as outlined in (A). One day prior to drug treatment, cells (from one donor) were nucleofected with 30 pmol of either non-targeting control siRNA or INTS2 siRNA. One day after drug treatment, a portion of the cells was collected for western blot analysis (E) and densitometry conducted to quantify the knockdown efficiency of the targeted proteins normalized to the loading controls. Another portion of the cells were subjected to Pol II or IgG ChIP assays using normal rabbit IgG for control to quantify occupancy of the Nuc-1 region of HIV LTR (F). The ChIP-qPCR signals were normalized to input DNA. The remaining cells were treated with a combination of AZD4573 and SMAP-2 for one day, followed by activation with PMA/I in the presence or absence of 2 μM morphine for an additional day, and subsequently analyzed for GFP expression by flow cytometry (G). For images (D, F, and G), data are represented as mean ± SD from three technical replicates, asterisks denote levels of statistical significance compared with the DMSO groups calculated by two-tailed Student’s *t* test (∗: *p* < 0.05, ∗∗: *p* < 0.01, and ∗∗∗: *p* < 0.001).

Cocaine or morphine were not tested alone for their effect on HIV-GFP expression; however, neither compound significantly enhanced PMA/I-induced reactivation (Figure 6B, DMSO group; *p* = 0.14 and 0.12, respectively), indicating only modest modulatory effects under these conditions. Importantly, the inhibitory effect of the drug combination was largely preserved in the presence of 2 μM cocaine or morphine. Under cocaine treatment, both blocking and locking effects were robustly maintained (Figures 6B and 6C). Under morphine, the blocking effect persisted, but the locking effect was more variable (Figure 6C). Overall, morphine partially reduced but did not abolish the repressive activity of the combination.

ChIP assays showed that morphine reduced INTS11 occupancy at the HIV LTR across control, SMAP-2, and combination-treated conditions (Figure 6D), indicating impaired integrator recruitment. This was accompanied by increased Pol II loading (Figure 6F), consistent with reduced termination efficiency and impaired integrator-mediated Pol II clearance.^38^ These findings support a model in which morphine interferes with integrator-dependent repression at the HIV promoter. However, morphine was unable to further increase Pol II loading on the LTR when INTS2 was knocked down (Figures 6E and 6F), indicating that morphine may reduce the locking effect through an integrator-dependent pathway. Consistent with this, the attenuating effect of morphine on AZD4573 + SMAP-2 repression disappeared in INTS2-depleted cells (Figure 6G). These findings suggest that morphine modestly disrupts the locking function of SMAP-2 by interfering with integrator-mediated repression.

## Discussion

Building on recent findings that pharmacological activation of PP2A synergizes with CDK9 inhibition in cancer transcriptional regulation,^22^ we asked whether a similar strategy could be adapted to enforce durable HIV silencing. Our results show that the CDK9 inhibitor AZD4573 and the PP2A agonist SMAP-2 work together to repress HIV transcription. The two drugs may exert complementary effects on the transcription cycle, with CDK9 inhibition reducing elongation-associated phosphorylation of Pol II and PP2A activation promoting dephosphorylation and termination. These opposing enzymatic activities converge on shared substrates such as the Pol II C-terminal domain (CTD). The drug combination not only blocks HIV reactivation but also locks the provirus into a silent state that persists for several days after drug withdrawal. Importantly, the combination remains effective even in the presence of cocaine or morphine. Given the high prevalence of recreational drug use among people living with HIV,^36^ this robustness is highly relevant for clinical translation.

Several small molecules, including dCA,^39^ spironolactone,^40^ and mTOR inhibitors,^41^ have previously demonstrated HIV-suppressive activity, only very few achieving weeks of repression. These findings illustrate that pharmacologic transcriptional silencing of HIV is achievable but can be difficult to sustain, underscoring the need for strategies that reinforce silencing at multiple points in the transcription cycle to promote durability.

Our work provides such an approach. We show that dual inhibition of P-TEFb and activation of the PP2A-integrator complex cooperatively suppresses HIV transcription. ChIP analyses reveal that this combination reduces Pol II loading at the LTR while increasing repressive epigenetic marks such as H3K27me3, which are associated with long-term silencing.^42^^,^^43^ CDK9 inhibition alone did not achieve this outcome, consistent with prior reports that paused Pol II maintains chromatin accessibility and acts as a “bookmark” for future transcription.^44^^,^^45^ Thus, blocking elongation alone is insufficient; silencing requires simultaneous reinforcement of epigenetic repression and removal of Pol II.^38^

Tat-dependent transactivation likely represents an additional mechanistic layer influenced by the AZD4573 + SMAP-2 combination. Tat normally binds the TAR RNA stem-loop and recruits P-TEFb to the HIV promoter to stimulate productive elongation. Because AZD4573 directly inhibits the kinase activity of CDK9 and SMAP-2 promotes PP2A-associated dephosphorylation within the integrator complex, the combination may indirectly impair Tat function by destabilizing the productive elongation environment required for Tat-mediated transactivation. Our ChIP data showing reduced Pol II Ser2 phosphorylation and decreased Pol II occupancy at the HIV LTR are consistent with diminished P-TEFb activity at the promoter. In addition, the increased recruitment of integrator following SMAP-2 treatment may further antagonize Tat-driven elongation by favoring premature termination over productive transcriptional elongation. While our current study did not directly examine Tat binding to TAR or Tat occupancy at the LTR, the data support a model in which SMAP-2 functionally counteracts Tat-dependent transcription by reinforcing integrator-mediated repression and opposing P-TEFb activity.

This mechanism aligns with earlier studies showing that PP2A inhibition activates HIV transcription^46^ and that the PP2A-integrator axis regulates both termination and elongation.^22^^,^^24^ INTS12 knockout, which increases Pol II occupancy at the LTR and promotes reactivation,^31^ further underscores integrator’s role in restricting productive transcription. By targeting two complementary steps, P-TEFb-driven elongation and integrator-mediated termination, the AZD4573 + SMAP-2 combination achieves deeper repression than either agent alone. This strategy parallels the logic of combination ART, where targeting multiple points in the viral life cycle maximizes suppression. Similarly, combining orthogonal transcriptional interventions may be a key to establishing durable latency.

Cocaine and morphine partially challenged but did not abolish this block-and-lock effect. Cocaine allowed both blocking and locking to persist, whereas morphine weakened the locking effect while leaving short-term block intact. This partial interference may reflect cocaine and morphine’s ability to activate HIV transcription via the NF-κB and P-TEFb pathways.^47^^,^^48^ Strikingly, our data reveal that morphine may specifically target the integrator pathway, as it reduced INTS11 loading and increased Pol II loading at the LTR in a manner that mirrors *INTS2* knockdown. Despite this challenge, the combination of AZD4573 and SMAP-2 induced suppression of HIV reactivation that persisted after their withdrawal, highlighting both the robustness and mechanistic specificity of the block-and-lock strategy. These findings identify integrator as a key node through which cocaine and morphine can disrupt HIV transcriptional control.

In addition to these mechanistic studies, we examined the broader transcriptional consequences of AZD4573 + SMAP-2 treatment in primary CD4^+^ T cells. In resting CD4^+^ T cells, most differentially expressed genes were unannotated protein-coding transcripts or long non-coding RNAs, with *MYC* notably downregulated. In contrast, activated CD4^+^ T cells showed fewer transcriptional changes, although *HEXIM1* was reduced in the drug-treated condition. These results suggest that while the dual treatment enforces HIV silencing, it also perturbs host gene expression in a context-dependent manner, with potentially distinct consequences in resting versus activated T cells. Although we did not pursue functional follow-up of these differentially expressed genes, the findings suggest potential off-target effects that will need to be considered as block-and-lock strategies move toward translational development.

In summary, we demonstrate that simultaneous inhibition of P-TEFb and activation of the PP2A-integrator axis enforces HIV silencing through both transcriptional and epigenetic mechanisms. This dual targeting produces a block-and-lock effect that is durable, partially resilient to cocaine and morphine, and mechanistically grounded in complementary disruption of Pol II function. These findings establish proof-of-concept for combination silencing therapies and lay the foundation for future efforts that optimize drug regimens, delivery systems, and treatment durability. Ultimately, our work points toward block-and-lock strategies as a realistic path to long-term, drug-free HIV remission.

### Limitations of the study

This study has minor limitations. The locking effect was monitored for only 6 days; longer-term studies are required to determine whether silencing can be maintained for clinically meaningful periods. Cocaine and morphine experiments used a single concentration (2 μM) and were conducted *in vitro*, which may not fully reflect *in vivo* exposures. In addition, while AZD4573 was selected for its potency and specificity, other CDK9 inhibitors should be evaluated to compare efficacy and safety across this drug class. Because the commercially obtained LRS chambers were provided without donor demographic information, the potential effects of donor age, sex, gender, race, and ethnicity could not be evaluated, limiting assessment of demographic sources of biological variability and the generalizability of the findings. We did not directly measure integrator-mediated nascent RNA cleavage; therefore, the effects of INTS2 depletion on the endonuclease function of the complex and the precise contribution of INTS11-mediated cleavage to SMAP-2-dependent repression remain unresolved. Finally, the average sequencing depth in our RNA-seq data were ∼4.5 million reads per sample, which is sufficient to capture robust transcriptional differences but may underestimate more subtle changes, particularly in low-abundance transcripts. Thus, while the RNA-seq data provides a useful control for assessing potential off-target effects, deeper sequencing would be required for comprehensive discovery.

## Resource availability

### Lead contact

Further information and requests for resources and reagents should be directed to and will be fulfilled by the lead contact, Melanie Ott (melanie.ott@gladstone.ucsf.edu).

### Materials availability

This study did not generate new unique reagents.

### Data and code availability

• The RNA-seq data generated in this study have been deposited in the NCBI Gene Expression Omnibus (GEO) and are publicly available under accession number GSE342431.
• This paper does not report original code.
• Any additional information required to reanalyze the data reported in this paper is available from the lead contact upon request.

## Acknowledgments

We thankfully acknowledge support from the Gladstone Institutes and the HOPE Collaboratory. We thank John C.W. Carroll for graphics arts, Irene Chen and Françoise Chanut for critical reading and editing of the manuscript, Veronica Fonseca and Kanika Khanna for administrative assistance. This research was supported by: 10.13039/100000002NIH awards R01DA063159, R01AI183286, R01DA059551, and R37AI083139; UM1AI164559, co-funded by 10.13039/100000050NHLBI, 10.13039/100000026NIDA, 10.13039/100000025NIMH, 10.13039/100000065NINDS, and 10.13039/100000062NIDDK. Gladstone Flow Cytometry Facility is supported by NIH S10 RR028962. We also acknowledge the generous support from James B. Pendleton Charitable Trust, Michael Hulton Center for HIV Cure Research, P. and E. Taft, and the Gordon and Betty Moore Foundation. M.O. is a Biohub, San Francisco, Investigator and the Nick and Sue Hellmann Distinguished Professor. The funders had no role in study design, data collection and analysis, decision to publish, or preparation of the manuscript.

## Author contributions

Conceptualization: Z.L. and M.O.; methodology: Z.L., F.J.Z.-B., and M.O.; investigation: Z.L., F.J.Z.-B., and P.G.; resources: Z.L. and M.O.; writing: Z.L., F.J.Z.-B., P.G., and M.O.; supervision: Z.L. and M.O.; funding acquisition: Z.L. and M.O.

## Declaration of interests

M.O. is a cofounder of DirectBio, Inc and on the SAB for Invisishield Technologies LTD.

## STAR★Methods

### Key resources table

**Table:** 

REAGENT or RESOURCE	SOURCE	IDENTIFIER
**Antibodies**		

mouse-anti-human INTS2	Santa Cruz	sc-514945
rabbit-anti-human Cyclin T1	Santa Cruz	sc-10750; RRID: AB_2073888
rabbit-anti-human CDK9	Cell Signaling	2316S; RRID: AB_2291505
mouse-anti-human α-tubulin	Sigma	CP06; RRID: AB_2617116
rabbit-anti-human INTS11	Bethyl	A301-274A; RRID: AB_937779
rabbit-anti-human INTS6	Bethyl	A301-658A; RRID: AB_1210927
rabbit-anti-human INTS8	Bethyl	A300-269A; RRID: AB_2280498
rabbit-anti-human INTS4	Bethyl	A301-296A; RRID: AB_937909
rabbit-anti-human PSMD3	Bethyl	A303-826A; RRID: AB_2621271
rabbit-anti-human FTSJ3	Bethyl	A304-199A; RRID: AB_2620396
rabbit-anti-human HEXIM1	Bethyl	A303-113A; RRID: AB_10892626
goat-anti-rabbit-680 nm	Thermo Fisher	A-21076; RRID: AB_2535736
goat-anti-mouse 680 nm	Thermo Fisher	A-21057; RRID: AB_141436
control rabbit IgG	Bethyl	P120-101; RRID: AB_479829
rabbit anti-Pol II	Bethyl	A300-653A; RRID: AB_519334
rabbit anti-H3K9me3	Active Motif	39765; RRID: AB_2793334
rabbit anti-H3K27me3	Active Motif	39155; RRID: AB_2561020
rabbit anti-Ac-H3	Millipore	06–599; RRID: AB_2115283
rabbit anti-Ac-H4	Millipore	06–598; RRID: AB_2295074
rabbit anti-H3K4me3	Active Motif	39159; RRID: AB_2615077
rabbit anti-Pol II Ser2p	Bethyl	A300-654A; RRID: AB_519341
rabbit anti-Pol II Ser5p	Abcam	ab5131; RRID: AB_449369
anti-CD28 antibody	BD Biosciences	555725; RRID: AB_396068
anti-CD3 antibody	BD Biosciences	555336; RRID: AB_395742
FITC-conjugated mouse anti-Human CD25 antibody	BD Biosciences	555431; RRID: AB_395825
PE-conjugated mouse anti-Human CD69 antibody	BD Biosciences	555531; RRID: AB_395916

**Bacterial and virus strains**		

HIV pNL4-3-Δ6-drEGFP	Yang et al.^48^	N/A

**Biological samples**		

Concentrated Human Blood in LRS Chambers	Vitalant	RE201

**Chemicals, peptides, and recombinant proteins**		

AZD4573	Selleckchem	S8719
SMAP-2	MedChemExpress	HY-120272
SNS-032	Selleckchem	S1145
Phorbol 12-myristate 13-acetate (PMA)	Selleckchem	S7791
Ionomycin	Selleckchem	S7074
N-hydroxysuccinimidyl ester (CFSE)	Abcam	ab113853
Protein A agarose	Invitrogen	15918–014
protease inhibitor cocktail	Sigma	P8340
JetPrime reagent	Sartorius	101000001
Poly-D-Lysine	Gibco	A3890401
DMEM medium	Corning	10-013-CV
RPMI medium	Corning	10-040-CV
Fetal Bovine Serum	Avantor	89510-186
Penicillin-Streptomycin Solution	Corning	30-002-Cl
Annexin V	Sartorius	4641
DNA/RNA Shield	Zymo Research	R1100-250
TRIzol Reagent	Thermo Fisher	15596018
M-MLV Reverse Transcriptase	Promega	M1701
random primers	Thermo Fisher	48190011
siRNA Negative control	Thermo Fisher	4390843
siRNA targeting INTS2	Thermo Fisher	s33184
siRNA targeting FTSJ3	Thermo Fisher	s42146
siRNA targeting PSMD3	Thermo Fisher	s11393
siRNA targeting INTS11	Thermo Fisher	s29893
siRNA targeting INTS6	Thermo Fisher	s25482

**Critical commercial assays**		

DyNAmo HS SYBR Green qPCR kit	Thermo Fisher	F410L
ChIP-IT Express Enzymatic kit	Active Motif	53009
SE Cell Line 4D-Nucleofector X Kit L	Lonza	V4XC-1024
Lymphoprep density gradient medium	STEMCELL	18061
EasySep Human CD4^+^ T Cell Isolation Kit	STEMCELL	17952
LIVE/DEAD Cell Stain Kit	Invitrogen	L34955
Zombie Aqua Fixable Viability Kit	BioLegend	423101

**Deposited data**		

Transcriptomic effects of combined AZD4573 and SMAP-2 treatment in resting and anti-CD3/CD28-activated primary human CD4^+^ T cells	Gene Expression Omnibus (GEO) Repository	GSE: 342431

**Experimental models: Cell lines**		

HEK 293T cells	ATCC	CRL-3216
J-Lat A2 cells	Jordan et al.^49^	N/A
J-Lat 5A8 cells	Chan et al.^32^	N/A

**Oligonucleotides**		

HIV-nuc1-F	Elim Biopharm	5′-CTGGGAGCTCTCTGGCTAACTA-3′
HIV-nuc1-R	Elim Biopharm	5′-TTACCAGAGTCACACAACAGACG-3′
qActB-F	Elim Biopharm	5′-AGAGCTACGAGCTGCCTGAC-3′
qActB-R	Elim Biopharm	5′-AGCACTGTGTTGGCGTACAG-3′
qSLFN5-F	Elim Biopharm	5′-AGAGAATGGACTGCTTGGATG-3′
qSLFN5-R	Elim Biopharm	5′-CTGCTCTTTCAGACATTCCGA-3′
HIV LTR-F	Elim Biopharm	5′-GGGTCTCTCTGGTTAGACCAG-3′
HIV LTR-R-1	Elim Biopharm	5′-GGGTTCCCTAGTTAGCCAGAG-3′
HIV LTR-R-2	Elim Biopharm	5′-CTGCTAGAGATTTTCCACACTGAC-3′

**Software and algorithms**		

SynergyFinder 3.0	Ianevski et al.^50^	https://synergyfinder.fimm.fi
Flowjo	Waters Biosciences	https://flowjo.com/

### Experimental model and study participant details

#### Cell lines

We cultured HEK 293T cells (American Type Culture Collection [ATCC]) in DMEM medium (Corning 10-013-CV), and Jurkat-derived cells in RPMI medium (Corning 10-040-CV) in a 5% CO_2_ humidified incubator at 37°C. Both mediums were supplemented with 10% fetal bovine serum (FBS, Avantor 89510-186) and 100 units/mL penicillin and 100 μg/mL of streptomycin (Corning 30-002-Cl). For HEK 293T cells, we split them by either 1:10 or 1:5 whenever the cell density reached 90%. For Jurkat-derived cells, we constantly maintain the cell density at 0.1 million to 0.8 million per mL. All cell lines were routinely tested and confirmed to be mycoplasma-free.

#### Human samples

Blood cells in leukoreduction system (LRS) chambers from healthy donors were purchased from Vitalant (catalog no. RE201). Vitalant obtained donor consent permitting donated blood products to be used for research. The LRS chamber samples were provided to the investigators without direct identifiers or donor-linked demographic or clinical information, and the investigators had no access to a code key or any other means of re-identifying the donors. The research team did not recruit, interact with, or intervene with the donors. CD4^+^ T cells isolated from these samples were used as *ex vivo* host cells for *in vitro* HIV infection and drug-treatment experiments. Donor age, sex, gender, race, and ethnicity were not provided and therefore could not be evaluated.

#### Primary cell cultures

Blood from anonymous donors was subjected to PBMC and CD4^+^ T cell isolation as described below. The CD4^+^ T cells were then cultured in RPMI medium, which was further supplemented with 10% FBS, 100 units/mL of penicillin, and 100 μg/mL of streptomycin, in a 5% CO_2_ humidified incubator at 37°C. For activation of primary CD4^+^ T cells, tissue-culture plates were coated with anti-CD3 antibody (BD Biosciences, 555336) diluted to 10 μg/mL in PBS. Six-well plates received 1 mL per well, and 12-well plates received 0.5 mL per well. Plates were incubated either overnight at 4°C or for 2 h at 37°C. The coating solution was then removed, and primary CD4^+^ T cells were added at 1 × 10^6^ cells/mL in culture medium containing 1 μg/mL soluble anti-CD28 antibody (BD Biosciences, 555725).

### Method details

#### Cell line-based HIV latency reversal assay

The Jurkat-based HIV-1 latency model J-Lat A2 (previously generated by Verdin lab based on human CD4^+^ T cells Jurkat line^49^) were first treated in triplicates for 24 h with single or combinations of 0.1% DMSO, AZD4573 (Selleckchem S8719), SMAP-2 (DT-1154, MedChemExpress HY-120272), SNS-032 (Selleckchem S1145) in the presence or absence of PMA (Selleckchem S7791) and Ionomycin (Selleckchem S7074) at the various concentrations indicated in the paper, and then re-suspended in cold phosphate-buffered saline (PBS). Quantification of the GFP^+^ cells was conducted on a BD Bioscience LSR Fortessa X20 cytometer. The data were analyzed with the Flowjo software and plotted as bar graphs.

#### Combinatorial titration of AZD4573 and SMAP-2 using incucyte-based quantification

To evaluate the combinatorial effect of AZD4573 and SMAP-2 on HIV latency reversal, J-Lat A2 cells were seeded at 10,000 cells per well in Poly-D-Lysine (Gibco A3890401) - coated 96-well plates and treated with a matrix of drug combinations. AZD4573 was tested across 12 concentrations ranging from 0 to 10 nM (1:2 serial dilutions), and SMAP-2 from 0 to 10 μM (1:3 serial dilutions). Cells were pretreated with drug combinations for 2 h before stimulation with 81 nM PMA + 0.1 μM Ionomycin in combination with Annexin V (Sartorius 4641). GFP expression and Annexin V staining were monitored every hour for 24 h using the Incucyte live-cell analysis system. The optimal exponential reactivation time point was identified (16 h post reactivation) and used to calculate synergistic interactions with the online software SynergyFinder 3.0 (https://synergyfinder.fimm.fi/).^50^ Synergy was calculated using the ZIP method with base-line correction to the drug matrix to identify concentration pairs that suppressed HIV reactivation without inducing cytotoxicity.

#### Latency reversal assay in latently infected primary CD4^+^ T cells

Blood cells in leukoreduction system (LRS) chambers from healthy volunteers were purchased from Vitalant (https://www.vitalant.org), and peripheral blood mononuclear cells (PBMCs) were isolated using Lymphoprep density gradient medium (STEMCELL 18061). CD4^+^ T cells were isolated from PBMCs using negative selection with EasySep Human CD4^+^ T Cell Isolation Kit (STEMCELL 17952) according to the manufacturer’s protocols and cultured at 10^7^ cells/mL in RPMI medium supplemented with 10% FBS, 100 units/mL penicillin and 100 μg/mL of streptomycin in a 5% CO_2_ humidified incubator at 37°C, or frozen in liquid nitrogen and then thawed in RPMI 1 hour before the subsequent experiments.

The construction of the primary cell latency model was based on the Greene model^32^^,^^51^ using a pNL4-3-Δ6-drEGFP construct^52^ obtained as a kind gift from the Siliciano lab. Specifically, Reporter virus was produced by transfecting 20 million HEK293T cells with 20 μg pNL4-3-Δ6-drEGFP using JetPrime (Sartorius 101000001). Viral supernatants were collected at 72 h post transfection, filtered through 0.45 μm filter, and spinoculated into 20 million resting primary CD4^+^ T cells (800 g, 30°C, 2 h). Cells were then switched to virus-free medium and cultured at 1 million/mL for 3 days before drug treatment.

Three days after viral infection, we treated the cells with 0.1% DMSO for control, 5 nM AZD4573, 5 μM SMAP-2, and their combination. We let the cells culture with the drugs for one day and proceeded to treat them with T-cell activation agents the next day. Again, we let these cells culture for another day and finished our procedure with a Zombie stain and analyzed by flow cytometry to check for GFP^+^ % and live cell %. To test for the “locking effect”, we changed the cells to fresh medium after the 1-day drug treatment and kept them in drug-free medium for 6 days, taking aliquots of the cells on days 3, 5, 7 and then subjected them to cell activation and flow cytometry as above to see how the drugs’ HIV-inhibiting would change over time.

#### Nucleofection in jurkat-based cells

A 4D-Nucleofector X Unit (Lonza AAF-1002X) was used for all experiments. For every 10^6^ Jurkat cells, 30 pmol siRNA targeting nothing (Thermo 4390843), INTS2 (Thermo s33184), FTSJ3 (Thermo s42146), PSMD3 (Thermo s11393), INTS11 (Thermo s29893), INTS6 (Thermo s25482) or their 1:1 combinations (15 pmol/each siRNA) were introduced using SE Cell Line 4D-Nucleofector X Kit L (Lonza V4XC-1024) according to the manufacturer’s protocol with the program CL-120. Two days after nucleofection, the cells were subjected to either FACS, drug treatment or Western blot assays.

#### Co-immunoprecipitation (Co-IP) assay

Approximately 4 × 10^7^ HEK 293T cells (from American Type Culture Collection) in two 150-mm dishes were transfected with siRNAs (2 μmol/dish) targeting INTS2 (ThermoFisher s33184) or nothing (ThermoFisher 4390843) using the JetPrime reagent (Sartorius Cat. 101000001). The following operations were all carried out at 4°C or on ice unless stated otherwise. Two days after transfection, the cells were harvested and swollen in 10 mL hypotonic buffer A [10 mM HEPES-KOH (pH 7.9), 1.5 mM MgCl_2_ and 10 mM KCl] for 5 min and then centrifuged at 362 g for 5 min. The cells were then resuspended in 5 mL buffer A and disrupted by grinding 20 times with a Dounce tissue homogenizer (Fisher K885300-0007), followed by centrifugation at 3,220 g for 10 min to collect the nuclei in a 15 mL Falcon tube. The nuclei were then extracted with an approximately equal volume of hypertonic buffer C [20 mM HEPES-KOH (pH 7.9), 0.3 M NaCl, 25% glycerol, 0.2 mM EDTA, 1.5 mM MgCl_2_, 0.4% NP-40, 1 mM dithiothreitol and 1 × protease inhibitor cocktail (Sigma P8340)] for 30 min and then centrifuged at 20,800 g for 10 min to obtain the supernatant (nuclear extracts or NE). For INTS11 IP, the NE was mixed with 2 μg INTS11 antibody (Bethyl A301-274A) or normal IgG for control (Bethyl P120-101) and 15 μL Protein A agarose (Invitrogen 15918–014). All IP reactions were rotated at 4°C overnight. After being washed three times with 1 mL buffer D [20 mM HEPES-KOH (pH 7.9), 0.15 M KCl, 15% glycerol, 0.2 mM EDTA and 0.4% NP-40], the agarose beads were eluted with 30 μL 0.1 M glycine-HCl (pH 2.0) at room temperature for 15 min. For Western blot, 2% of the NE input and 50% of the IP eluate from each group were loaded into each NE and IP lane, respectively.

#### Chromatin immunoprecipitation (ChIP) assay

Primary resting CD4^+^ T cells were fixed with 1% formaldehyde for 10 min at room temperature, and ChIP assays were performed using the ChIP-IT Express Enzymatic kit (Active Motif 53009) according to the manufacturer’s protocol. For each ChIP reaction, 2 μg of the following antibodies were used: control rabbit IgG (Bethyl P120-101), rabbit anti-Pol II (Bethyl A300-653A), rabbit anti-H3K9me3 (Active Motif 39765), rabbit anti-H3K27me3 (Active Motif 39155), rabbit anti-Ac-H3 (Millipore 06–599), rabbit anti-Ac-H4 (Millipore 06–598), rabbit anti-H3K4me3 (Active Motif 39159), rabbit-*anti*-human INTS11 (Bethyl A301-274A), rabbit anti-Pol II Ser2p (Bethyl A300-654A), rabbit anti-Pol II Ser5p (Abcam ab5131). qPCR was performed with the primers HIV-nuc1-F (5′-CTGGGAGCTCTCTGGCTAACTA-3′) and HIV-nuc1-R (5′-TTACCAGAGTCACACAACAGACG-3′). Signals obtained by qPCR were normalized to those of input DNA, and the averages from triplicate qPCR reactions were used to generate standard deviation depicted by error bars. Two-tailed Student’s *t*-tests were conducted, and the different significance levels were marked by 1–3 asterisks (∗: *p* < 0.05, ∗∗: *p* < 0.01, and ∗∗∗: *p* < 0.001).

#### Reverse transcription and quantitative PCR (qRT-PCR) assay

Total cellular RNAs were extracted by using TRIzol Reagent (Thermo Fisher 15596018). After quantification using NanoDrop, 2 μg total RNA was reverse transcribed by M-MLV Reverse Transcriptase (Promega M1701) with random primers (Thermo Fisher 48190011). The cDNAs were then subjected to qPCR using DyNAmo HS SYBR Green qPCR kit (Thermo Fisher F410L) on a CFX384 system (Bio-Rad) with the primer pairs (0.5 μM): qActB-F (5′-AGAGCTACGAGCTGCCTGAC-3′)/qActB-R (5′-AGCACTGTGTTGGCGTACAG-3′), HIV-F (5′-GCCTCAATAAAGCTTGCCTTGA-3′)/HIV-R (5′-GGGCGCCACTGCTAGAGA-3′), and qSLFN5-F (5′-AGAGAATGGACTGCTTGGATG-3′)/qSLFN5-R (5′-CTGCTCTTTCAGACATTCCGA-3′). To distinguish short vs. long HIV transcript, the forward primer HIV LTR-F (5′-GGGTCTCTCTGGTTAGACCAG-3′) was paired with either HIV LTR-R-1 (5′-GGGTTCCCTAGTTAGCCAGAG-3′) or HIV LTR-R-2 (5′-CTGCTAGAGATTTTCCACACTGAC-3′).^53^ qPCR reactions were performed with an initial denaturation at 95°C for 5 min, followed by 40 cycles of 94°C for 10 s, 55°C for 30 s, and 72°C for 30 s, with fluorescence acquisition at the end of each extension step. Amplification was followed by melt-curve analysis to assess product specificity. All reactions were carried out in triplicates with 20 μL reaction per well. The PCR signals were normalized to those of β-actin using the 2^−ΔΔCt^ method and displayed as scatterplots with error bars representing standard deviation (for linear graphs) or standard error of the mean (for log graphs).

#### Western blot analyses

For Western blot, 0.2 million Jurkat-based cells were lysed in 20 μL Laemmli buffer and loaded per lane. Primary antibodies used for western blots were mouse-*anti*-human INTS2 (Santa Cruz sc-514945), rabbit-*anti*-human Cyclin T1 (Santa Cruz sc-10750), rabbit-*anti*-human CDK9 (Cell Signaling 2316S), and mouse-*anti*-human α-tubulin (Sigma CP06), rabbit-*anti*-human INTS11 (Bethyl A301-274A), rabbit-*anti*-human INTS6 (Bethyl A301-658A), rabbit-*anti*-human INTS8 (Bethyl A300-269A), rabbit-*anti*-human INTS4 (Bethyl A301-296A), rabbit-*anti*-human PSMD3 (Bethyl A303-826A), rabbit-*anti*-human FTSJ3 (Bethyl A304-199A), rabbit-*anti*-human HEXIM1 (Bethyl A303-113A). Secondary antibodies used for western blots were goat-*anti*-rabbit 680 nm (Thermo Fisher A-21076) and goat-*anti*-mouse 680 nm (Thermo Fisher A-21057). The primary antibodies were diluted to 1 μg/mL except the tubulin antibody (200 ng/mL). Secondary antibodies were diluted to 200 ng/mL. The original Western blot scans are included in Document S1.

#### CD4^+^ T cell activation, proliferation and viability assays

For T cell activation assays, CD4^+^ T cells were isolated from the blood of healthy donors, treated with 0.1% DMSO, the combination of 5 nM AZD4573 + 5 μM SMAP-2, or antibodies targeting CD3 (BD Biosciences 555336) and CD28 (BD Biosciences 555725) for 24 h, and then stained with PE-conjugated mouse anti-Human CD69 antibody (BD Biosciences, 555531) and FITC-conjugated mouse anti-Human CD25 antibody (BD Biosciences, 555431). Flow cytometry was conducted on a BD Bioscience LSR Fortessa X20 cytometer, and data were analyzed using the Flowjo software.

For proliferation assays, CD4^+^ T cells are isolated from the blood of healthy donors were treated with 10 μM 5(6)-carboxyfluorescein N-hydroxysuccinimidyl ester (CFSE, Abcam ab113853) for 15 min. The cells were then treated with 0.1% DMSO, the combination of 5 nM AZD4573 + 5 μM SMAP-2, or antibodies targeting CD3 and CD28 for 24 h, and then washed and cultured for another 4 days in fresh medium. The cells were then subjected to flow cytometry as above to quantify the cells with reduced levels of CFSE stains.

For viability assay, CD4^+^ T cells isolated from the blood of healthy donors were treated with 0.1% DMSO or the combination of 5 nM AZD4573 + 5 μM SMAP-2 for 3 days. On each day, an aliquot of cells from each treatment group was stained with LIVE/DEAD Cell Stain Kit (Invitrogen, L34955). Untreated cells were used for day 0. The cells were then subjected to flow cytometry as above to quantify the percentage of live cells.

#### RNA-seq in CD4^+^ T cells treated with AZD4573 and SMAP-2

Primary CD4^+^ T cells were isolated from three independent HIV-negative donors. For each donor, cells were either maintained without stimulation or activated for 48 h with plate-bound anti-CD3/CD28 antibodies. Resting and activated cells were subsequently treated for 24 h with vehicle control (0.1% DMSO) or a combination of AZD4573 (5 nM) and SMAP-2 (5 μM). For each condition, 100,000 cells in 50 μL DNA/RNA Shield (Zymo Research, R1100-250) and submitted to Plasmidsaurus for RNA extraction, library preparation, sequencing, and primary bioinformatic analysis. Total RNA was extracted by Plasmidsaurus using a bead-based extraction method. RNA concentration was measured using a fluorescence-based plate-reader assay, and samples were concentration-normalized before library preparation. Polyadenylated RNA was captured and reverse-transcribed using poly(dT)VN primers containing sample barcodes and unique molecular identifiers (UMIs). Following second-strand cDNA synthesis, the double-stranded cDNA was fragmented by tagmentation, amplified, and indexed using unique dual i5/i7 indices. Libraries were sequenced on an Illumina NovaSeq X Plus using a stranded, single-end 3′-end-counting configuration. Reads were approximately 90 bp long and were predominantly derived from the terminal approximately 400 nucleotides of polyadenylated transcripts. UMIs incorporated during cDNA synthesis were used to distinguish independently captured RNA molecules from PCR-derived duplicates. Base-call files were converted to FASTQ format and demultiplexed using BCL Convert v4.3.6 and fqtk v0.3.1. Reads were filtered with fastp v0.24.0, including poly-X-tail trimming, 3′ quality-based trimming, a minimum Phred quality score of 15, and a minimum retained read length of 50 bp. Filtered reads were aligned to the human reference genome GRCh38 using STAR v2.7, with removal of noncanonical splice-junction alignments. BAM files were coordinate-sorted with SAMtools v1.21. PCR and optical duplicates were removed on the basis of UMI sequences using UMICollapse v1.1.0. Alignment quality, strand specificity, and the distribution of reads across genomic features were evaluated using RustQC v0.2.1, and aggregate quality-control reports were generated using MultiQC v1.33. Gene-level counts were generated using featureCounts in the Subread package v2.1.1 with strand-specific counting. Multimapping reads were assigned fractionally, and reads assigned to exonic and 3′-untranslated-region features were grouped by gene_id according to the reference GTF annotation. Gene counts were annotated with gene biotype and other metadata from the reference annotation. Sample correlations and principal-component analyses were calculated from trimmed mean of M-values–normalized counts using Pearson correlation. Differential gene-expression analysis was performed using edgePython v0.2.5. Samples yielded an average of approximately 4.5 million raw reads per sample. Within each activation condition, AZD4573/SMAP-2-treated samples were compared with donor-matched DMSO controls. Volcano plots display the log2 fold change for each gene against its corresponding −log10 adjusted *p* value.

### Quantification and statistical analysis

Quantitative data are presented as the arithmetic mean ± standard deviation (SD) or mean ± standard error of the mean (SEM), as specified in the corresponding figure legends. Exact values of n, the definition of n as technical or biological replicates, statistical tests, comparison groups, and measures of dispersion are provided in the corresponding figure legends. Biological replicates refer to experiments performed using primary CD4^+^ T cells obtained from independent donors. Technical replicates refer to replicate wells, samples, or qPCR reactions derived from the same biological sample, as applicable. Unless otherwise indicated, cell-line-based flow cytometry, qRT-PCR, and ChIP-qPCR assays were analyzed using three technical replicates. Primary CD4^+^ T-cell block-and-lock experiments in Figure 3 used cells from three independent donors, RNA-seq experiments in Figures 5E and 5F used cells from three independent donors, and the cocaine and morphine experiments in Figures 6B and 6C used cells from four independent donors. Primary-cell experiments performed using one or two donors are identified as such in the respective figure legends.

Flow cytometry data were analyzed using FlowJo (Waters Biosciences). Statistical analyses and graph generation were performed using Microsoft Excel. Western-blot band intensities were quantified using ImageJ and normalized to the indicated loading controls. Unless otherwise indicated, pairwise comparisons were performed using two-tailed Student’s t-tests, with DMSO-treated samples serving as the reference group. All statistical tests were two-sided. Statistical significance was defined as *p* < 0.05. Asterisks denote ∗*p* < 0.05, ∗∗*p* < 0.01, and ∗∗∗*p* < 0.001; n.s. denotes *p* ≥ 0.05.

For pairwise combination experiments, the expected additive effect was calculated using the Bliss independence model:

$$f_{xy,p}=f_{x}+f_{y}-\left(f_{x}\times f_{y}\right)$$

where f_x_ and f_y_ represent the effects of the individual perturbations and f_xy,p_ represents the predicted additive effect of the combination. Synergy was calculated as Δf_xy_ = f_xy,o_ − f_xy,p_, where f_xy,o_ is the observed combination effect; positive Δf_xy_ values indicate synergy beyond the predicted additive effect. For the AZD4573–SMAP-2 concentration matrices, synergy was analyzed using SynergyFinder 3.0 with the zero-interaction potency (ZIP) model and baseline correction. The 16-h time point, corresponding to the exponential phase of HIV reactivation, was used for the Incucyte-based synergy analysis.

For RNA-seq, *n* = 3 represents primary CD4^+^ T cells obtained from three independent HIV-negative donors. Resting and anti-CD3/CD28-activated cells were analyzed separately, and AZD4573 plus SMAP-2-treated samples were compared with the corresponding donor-matched DMSO controls. Trimmed mean of M-values-normalized counts were used for principal-component analysis and Pearson sample-to-sample correlation analysis. Differential gene-expression analysis was performed using edgePython v0.2.5. Volcano plots display log2 fold change against −log10 adjusted *p* value.

For reporting the replicate structure and summary measures: Figures 1, 2, and 4 use mean ± SD from three technical replicates; Figure 3 uses mean ± SD across three donors; Figures 6B and 6C uses mean ± SEM across four donors; and Figures 6D, 6F, and 6G use mean ± SD from three technical replicates.

## Supplemental information

Document S1. Figures S1–S7

Table S1. Original Incucyte data

Table S2. Original RNA-seq results

Data S1. Original Western Blot Data Used in this Study
