# Supplementary material for: “Locking” CDK9 inhibition by activating integrator function in HIV transcription
Source: iScience. 2026 Oct 17;29(11):117894. doi: 10.1016/j.isci.2026.117894 (PMC13695381; doi:10.1016/j.isci.2026.117894)
Supplement: Supplementary file 1 [file mmc1.pdf]

iScience, Volume *29*

Supplemental information

"Locking" CDK9 inhibition by activating integrator function in HIV transcription

Zichong Li, Francisco J. Zapatero-Belinchón, Patrik Geleziunas, and Melanie Ott

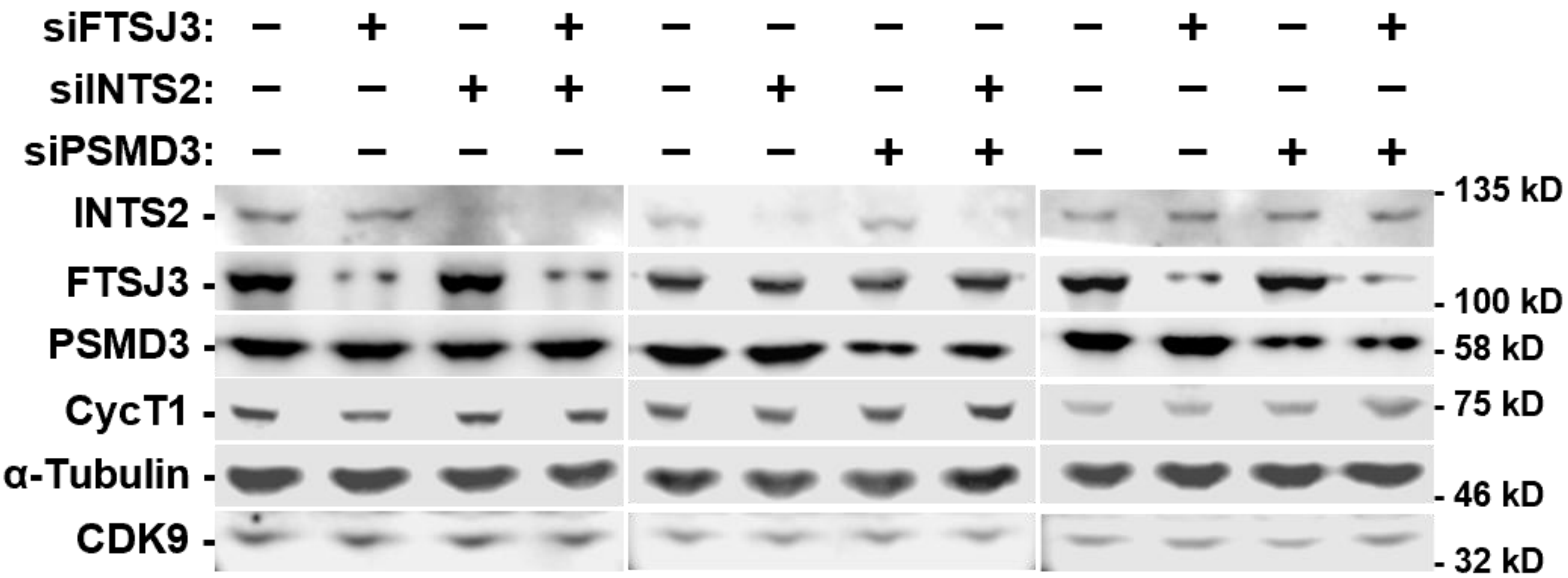

Figure S1. Western Blot Showing the siRNA Knockdown Efficiency in Figure 1B-1E. An aliquot of the cells used in experiments from Fig. 1B-1E were subjected to Western Blot for the indicated proteins.

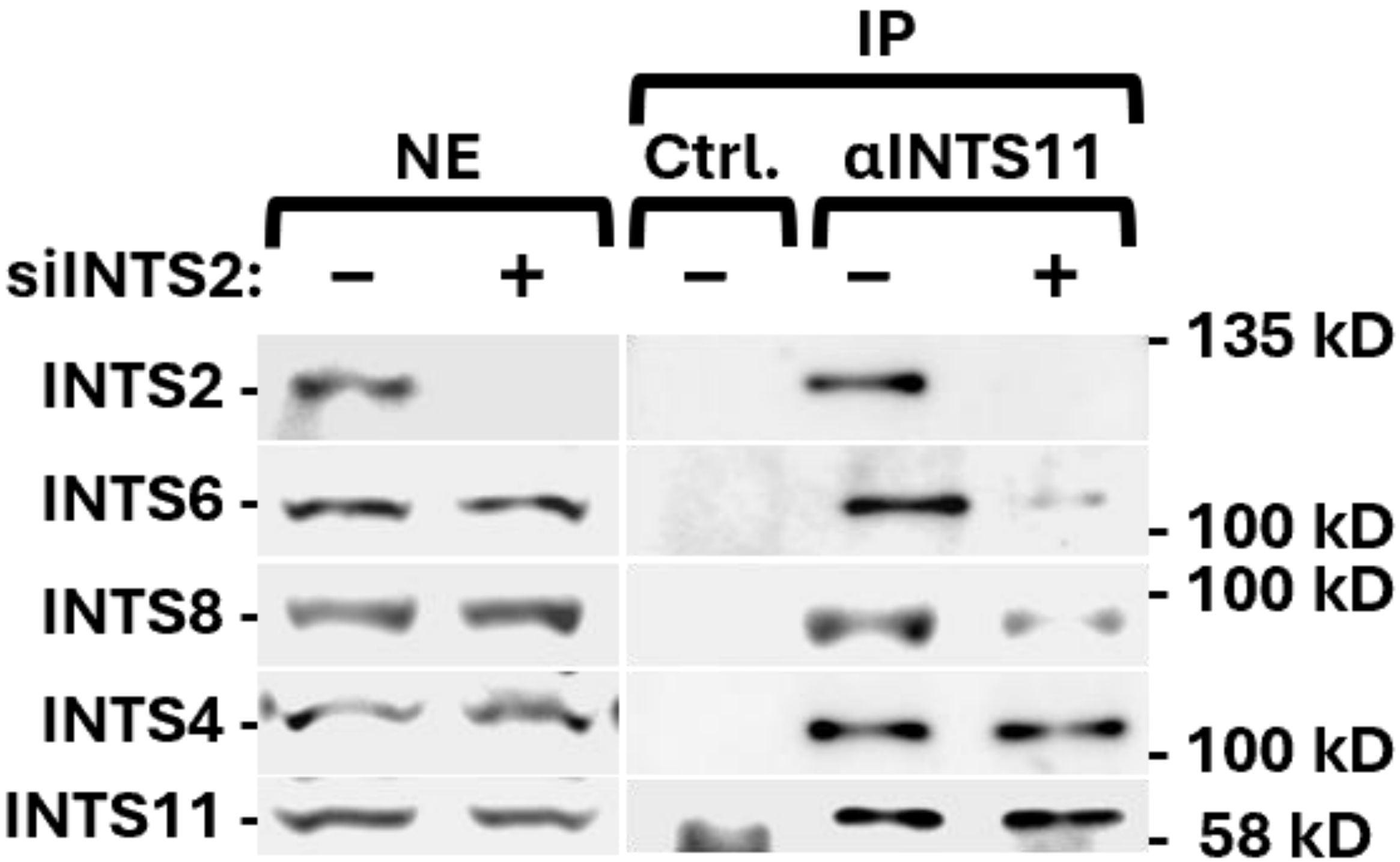

Figure S2. Co-IP Targeting INTS11 in HEK 293T Cells Transfected with Either Control siRNA or siINTS2, related to Fig. 1

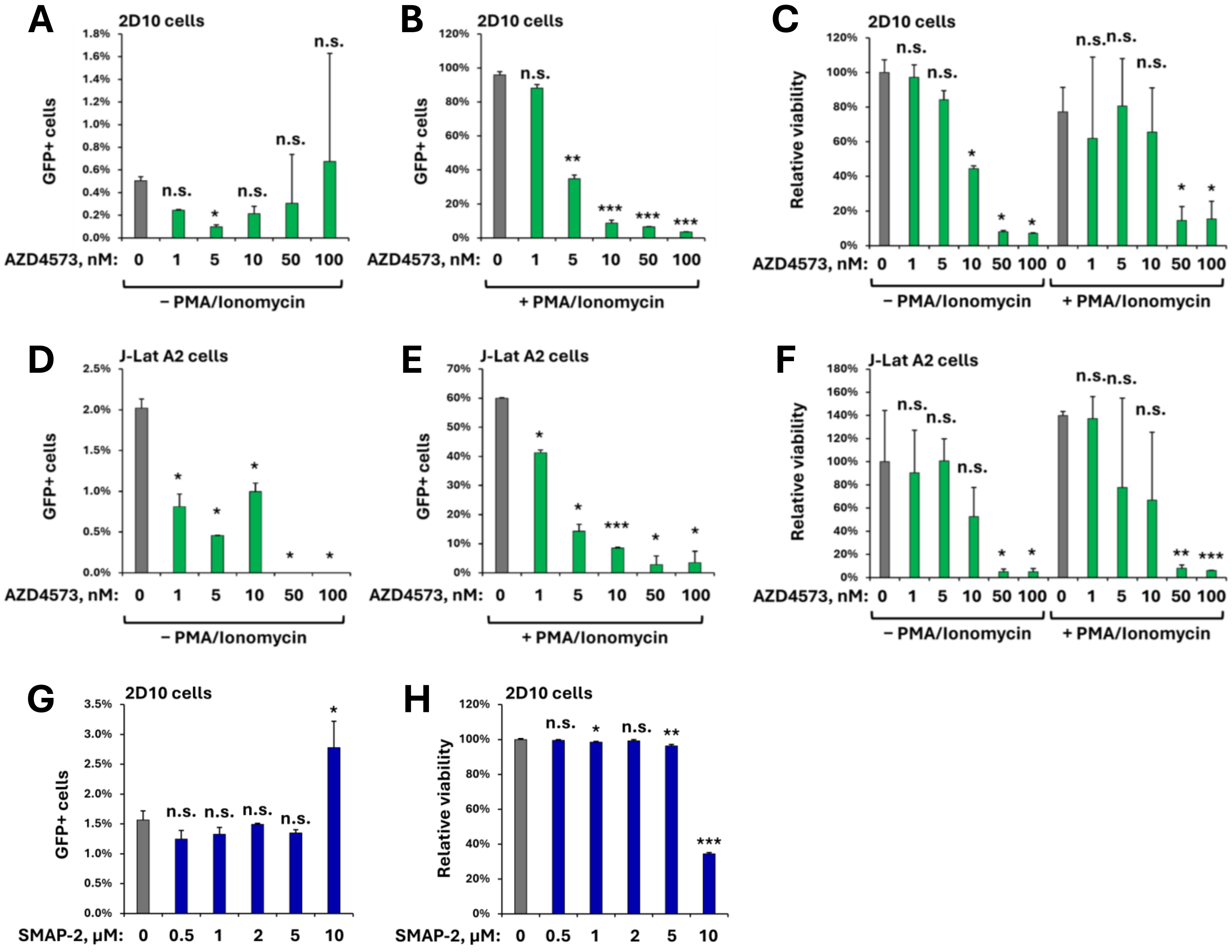

Figure S3. Inhibiting P-TEFb but not Activating PP2A Reduces HIV Reactivation in Cell Line-Based Latency Models, related to Fig. 1. J-Lat A2 or Jurkat 2D10 cells (as labelled on each panel) were treated with the indicated drugs at the indicated concentrations, with or without 25 nM PMA + 0.5 μM Ionomycin for 22 hours and subjected to flow cytometry (FACS) to assess GFP^{+} percentage (**A, B, D, E & G**) and viability (**C, F & H**). Data are represented as mean +/- SD from three technical replicates. Asterisks denote levels of statistical significance compared with the DMSO groups calculated by two-tailed Student's t-test (*: $p < 0.05$, **: $p < 0.01$, and ***: $p < 0.001$).

A

GFP+:	DMSO	AZD4573, nM: 0.01	AZD4573, nM: 0.02	AZD4573, nM: 0.04	AZD4573, nM: 0.08	AZD4573, nM: 0.156	AZD4573, nM: 0.313	AZD4573, nM: 0.625	AZD4573, nM: 1.25	AZD4573, nM: 2.5	AZD4573, nM: 5	AZD4573, nM: 10
DMSO	35.90%	45.90%	35.40%	34.70%	32.70%	33.50%	27.20%	24.10%	22.00%	20.40%	13.00%	3.62%
SMAP-2, nM: 13.7	36.10%	32.20%	30.70%	30.90%	**28.70%**	**28.50%**	37.70%	**20.40%**	**16.70%**	**17.10%**	**11.90%**	4.93%
SMAP-2, nM: 41	34.60%	39.00%	31.80%	30.70%	35.70%	**26.20%**	25.80%	25.00%	**16.80%**	**14.10%**	**11.80%**	3.42%
SMAP-2, nM: 123	36.00%	32.10%	33.60%	31.40%	**29.70%**	**27.20%**	28.00%	**21.60%**	26.70%	**14.00%**	**11.60%**	4.36%
SMAP-2, nM: 370	38.70%	34.60%	32.70%	**27.80%**	**29.90%**	**27.20%**	**25.80%**	**22.40%**	**20.40%**	**16.20%**	12.30%	3.28%
SMAP-2, nM: 1,111	36.60%	35.20%	33.00%	37.40%	31.40%	**27.50%**	26.10%	**19.70%**	**18.90%**	**16.60%**	22.60%	3.06%
SMAP-2, nM: 3,333	40.50%	35.20%	34.90%	33.30%	30.40%	28.10%	26.50%	**21.20%**	**18.10%**	**15.30%**	**11.00%**	2.57%
SMAP-2, nM: 10,000	48.20%	44.40%	45.50%	42.50%	44.30%	42.30%	36.90%	32.70%	30.40%	26.70%	13.70%	2.02%

B

Viability:	DMSO	AZD4573, nM: 0.01	AZD4573, nM: 0.02	AZD4573, nM: 0.04	AZD4573, nM: 0.08	AZD4573, nM: 0.156	AZD4573, nM: 0.313	AZD4573, nM: 0.625	AZD4573, nM: 1.25	AZD4573, nM: 2.5	AZD4573, nM: 5	AZD4573, nM: 10
DMSO	64.10%	66.30%	67.10%	71.20%	68.80%	63.40%	68.90%	69.50%	70.30%	69.70%	73.00%	74.80%
SMAP-2, nM: 13.7	69.60%	69.30%	72.00%	77.70%	70.80%	70.90%	70.30%	68.00%	69.30%	72.20%	78.20%	72.70%
SMAP-2, nM: 41	69.90%	71.20%	71.10%	69.70%	70.10%	70.70%	71.00%	70.80%	69.30%	70.80%	72.50%	75.10%
SMAP-2, nM: 123	69.50%	70.10%	72.10%	70.70%	68.80%	70.90%	69.50%	69.50%	70.80%	71.50%	73.50%	74.10%
SMAP-2, nM: 370	69.40%	68.70%	70.80%	70.20%	69.80%	71.00%	71.50%	70.90%	69.20%	72.20%	74.10%	74.60%
SMAP-2, nM: 1,111	68.60%	70.90%	71.20%	71.70%	71.70%	73.20%	71.60%	70.20%	71.60%	72.70%	73.80%	74.50%
SMAP-2, nM: 3,333	70.10%	71.80%	71.20%	70.50%	72.80%	71.80%	72.10%	72.00%	72.20%	73.50%	73.10%	74.60%
SMAP-2, nM: 10,000	57.10%	57.70%	59.10%	59.70%	59.00%	58.90%	60.60%	60.40%	61.70%	60.30%	58.60%	53.10%

Figure S4. Flow Cytometry Validation of AZD4573–SMAP-2 Synergy in Suppressing HIV Reactivation, related to Fig. 1. J-Lat A2 cells (n = 1) were treated with a concentration matrix of AZD4573 (0–10 nM) and SMAP-2 (0–10 μM) for 2 hours, followed by stimulation with 81 nM PMA and 0.1 μM ionomycin in the continued presence of the drugs. GFP expression (**A**) and cell viability assessed by FSC/SSC gating (**B**) were analyzed 24 hours post-treatment by flow cytometry. Concentration pairs that synergistically reduced the percentage of GFP^+ cells based on the Bliss independence model are highlighted in panel A.

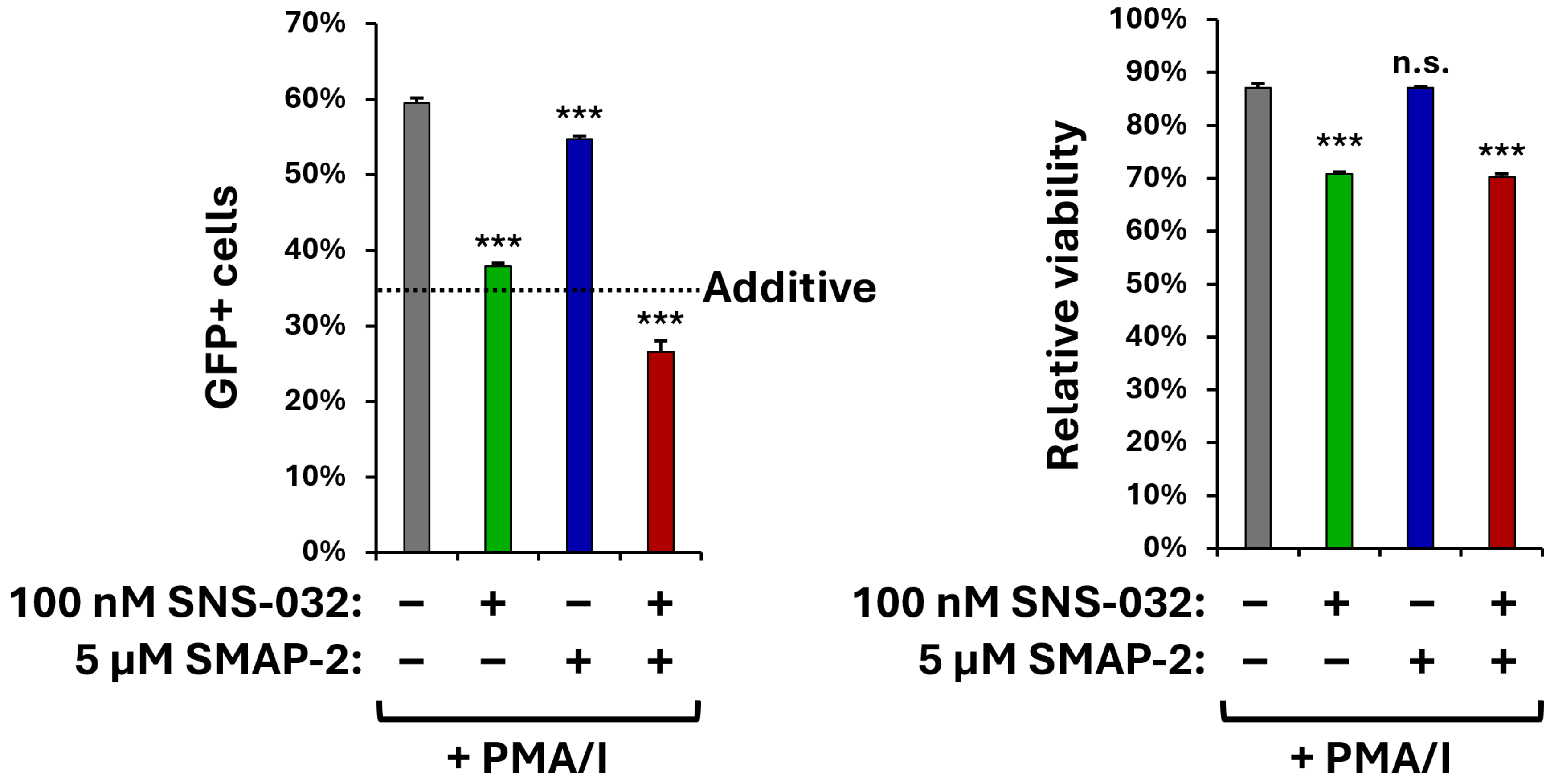

Figure S5. Synergistic Suppression of PMA/Ionomycin-induced HIV Reactivation by SNS-032 and SMAP-2 in J-Lat Cells, related to Fig. 1. J-Lat 5A8 cells were treated with 100 nM SNS-032, 5 µM SMAP-2, their combination, or DMSO for 2 hours and then 25 nM PMA + 0.5 µM Ionomycin were added for 1 day. The cells were then subjected to flow cytometry to quantify GFP^+ cells and relative viability (normalized to the DMSO group). The predicted level of additive, based on the Bliss Independence Model is labeled on the GFP panel. Data are represented as mean +/- SD from three technical replicates. Asterisks denote levels of statistical significance compared with the DMSO groups calculated by two-tailed Student's t-test (*: $p < 0.05$, **: $p < 0.01$, and ***: $p < 0.001$).

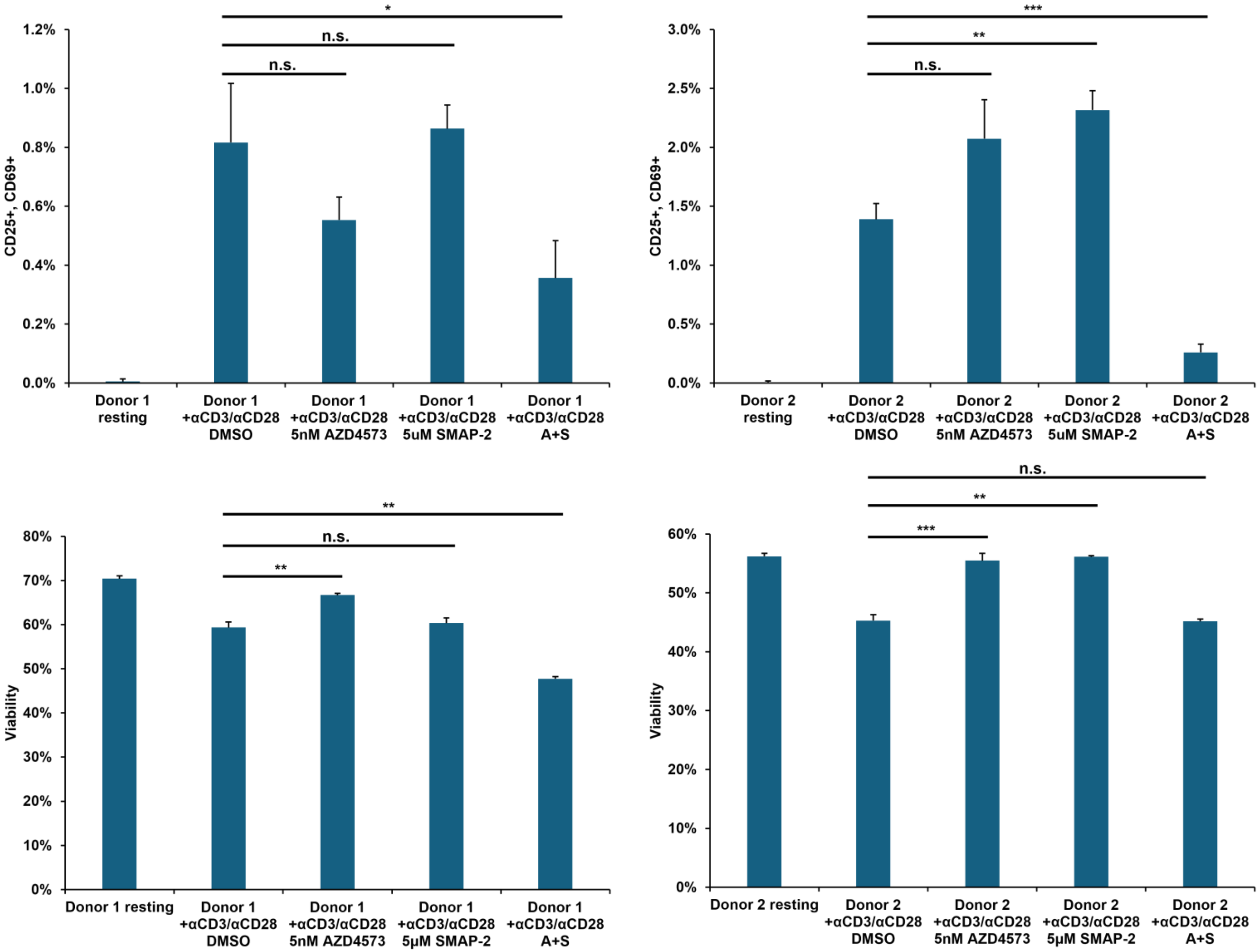

Figure S6. Quantification of CD69 and CD25 Expression in Activated Primary CD4⁺ T Cells from Two Donors, related to Fig. 5. Primary CD4⁺ T cells from n = 2 donors were treated with DMSO, 5 nM AZD4573, 5 µM SMAP-2, or their combination in the presence of anti-CD3/CD28 stimulation for 24 hr. Cell surface expression of the T cell activation markers CD69 and CD25 was quantified by immunostaining and flow cytometry. The graphs represent quantification of the flow cytometry data shown in Figure 5B. Data are represented as mean +/- SD from three technical replicates for each donor. Statistical significance relative to the DMSO group was determined by two-tailed Student's t-test (*: $p < 0.05$; **: $p < 0.01$; ***: $p < 0.001$).

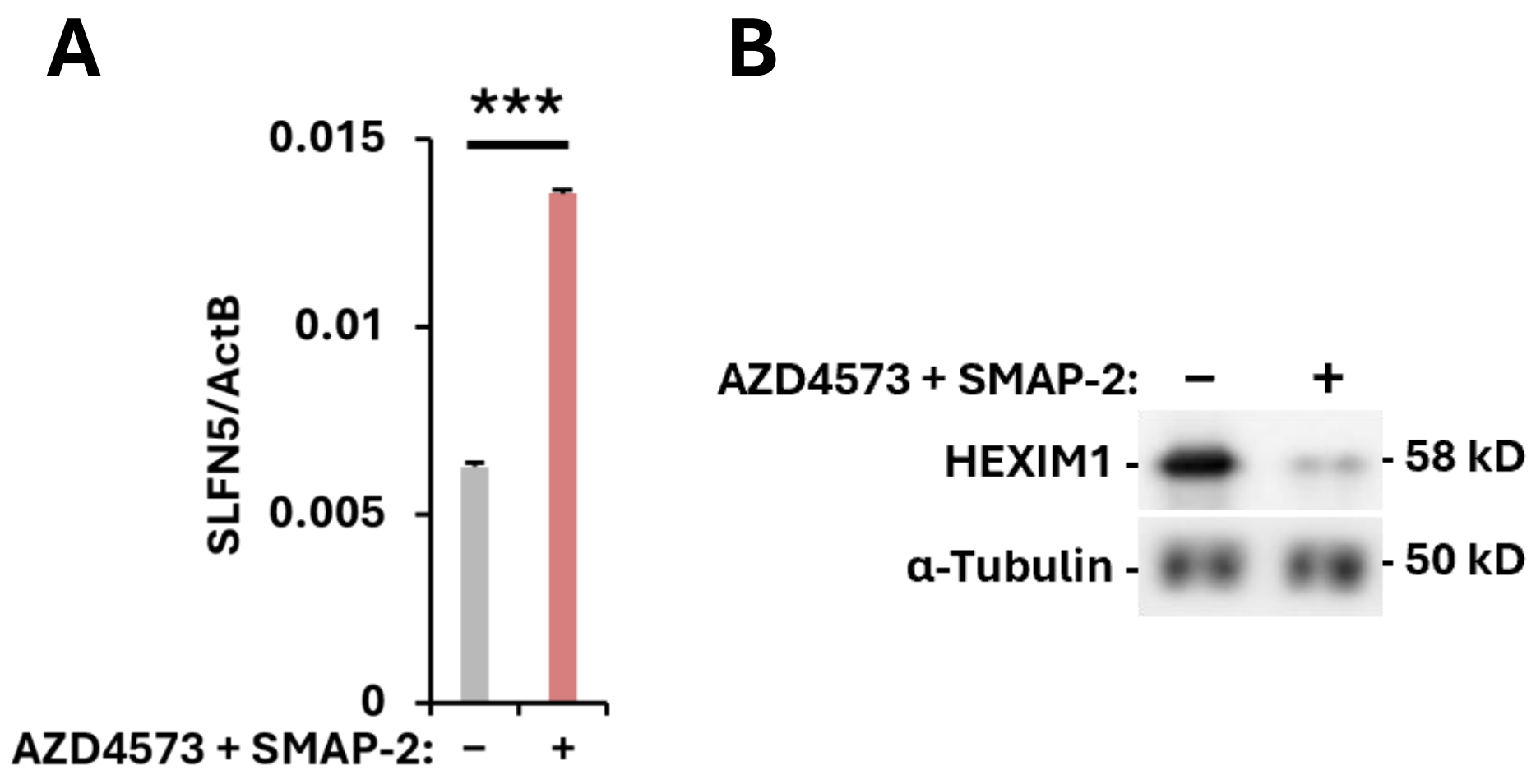

Figure S7. Validation of two Differentially Expression Genes, *SLFN5* by qRT-PCR (A), and *HEXIM1* by Western Blot (B), from Activated Primary CD4 T Cells (n = 1), related to Fig. 5. In panel A, the qRT-PCR data are represented as mean +/- SD from three technical replicates. Asterisks denote levels of statistical significance compared with the DMSO group calculated by two-tailed Student's t-test (*: $p < 0.05$, **: $p < 0.01$, and ***: $p < 0.001$).
