# Supplementary material for: “Locking” CDK9 inhibition by activating integrator function in HIV transcription
Source: iScience. 2026 Oct 17;29(11):117894. doi: 10.1016/j.isci.2026.117894 (PMC13695381; doi:10.1016/j.isci.2026.117894)

DATA S1. Original Western Blot Data Used in this Study

Original West Blot scans for Fig. S1

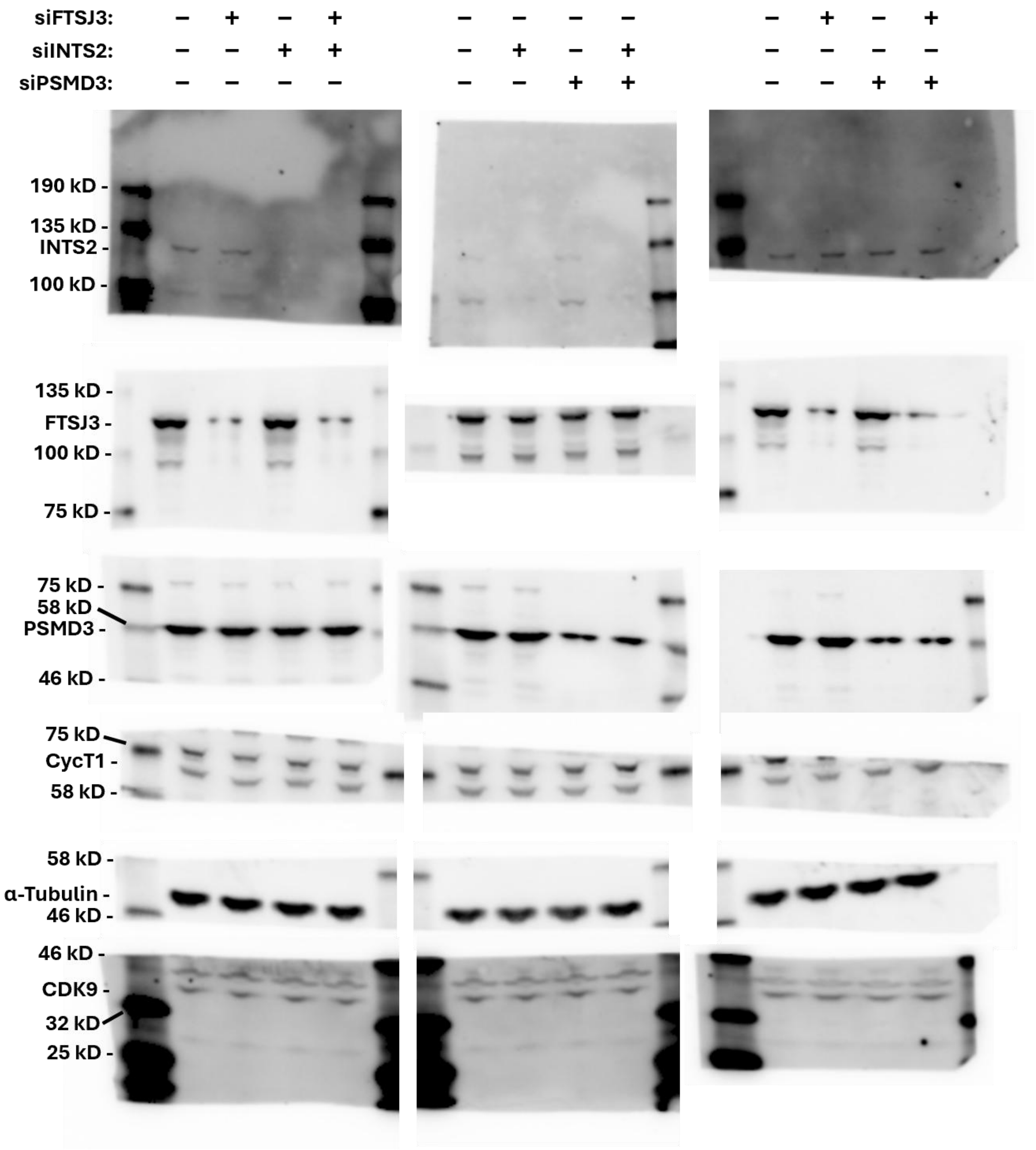

Original West Blot scans for Fig. S2

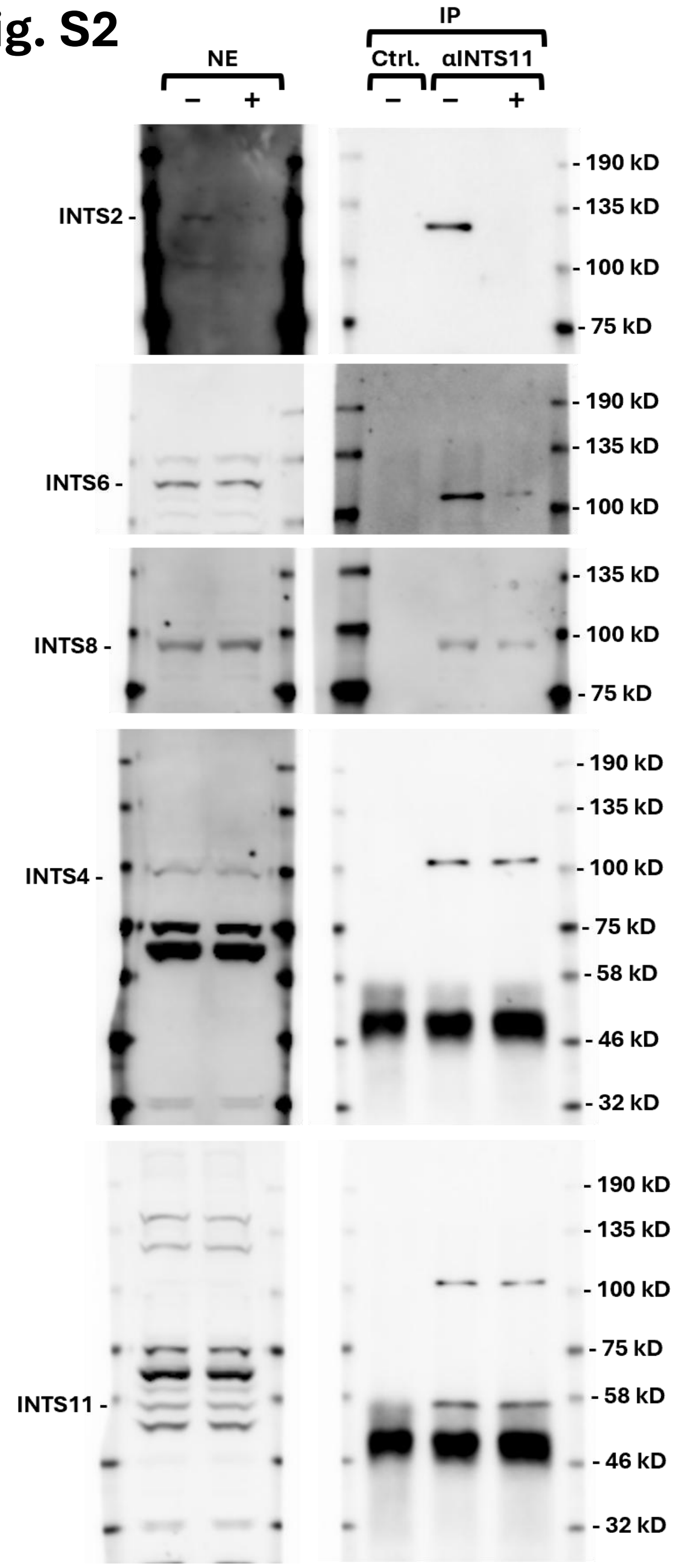

Original West Blot scans for Figs. 2D, 2E, 2F

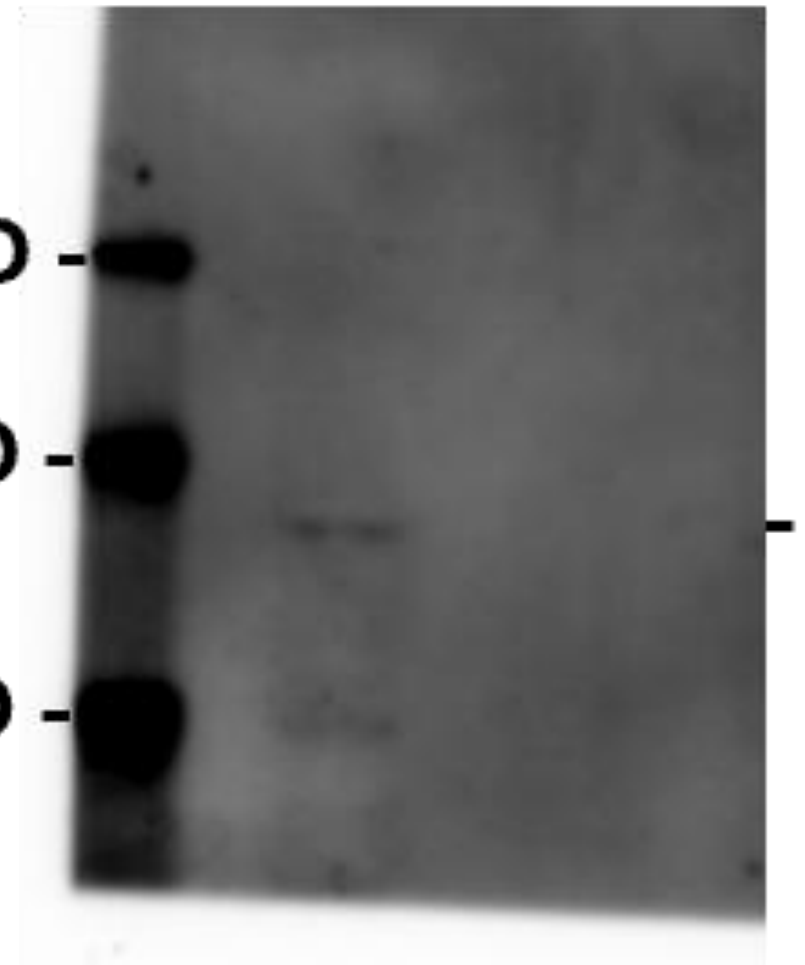

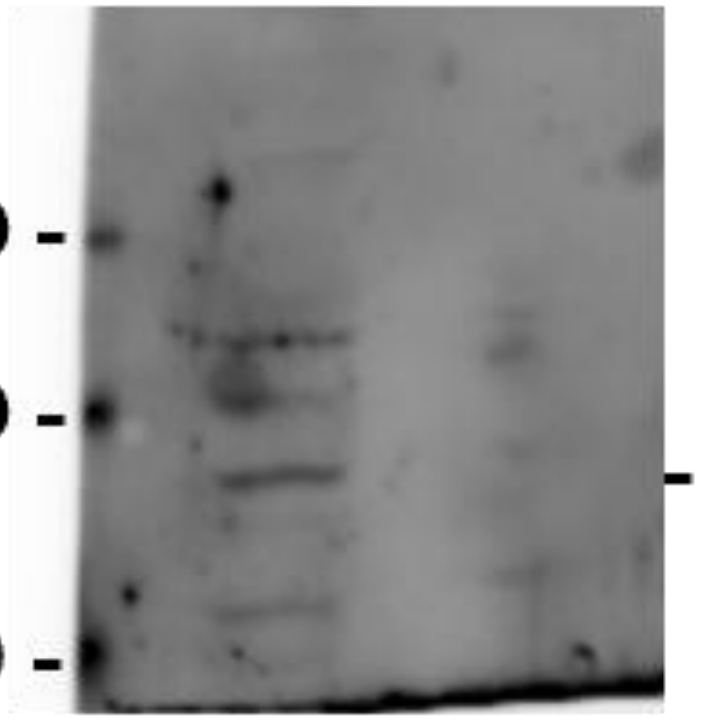

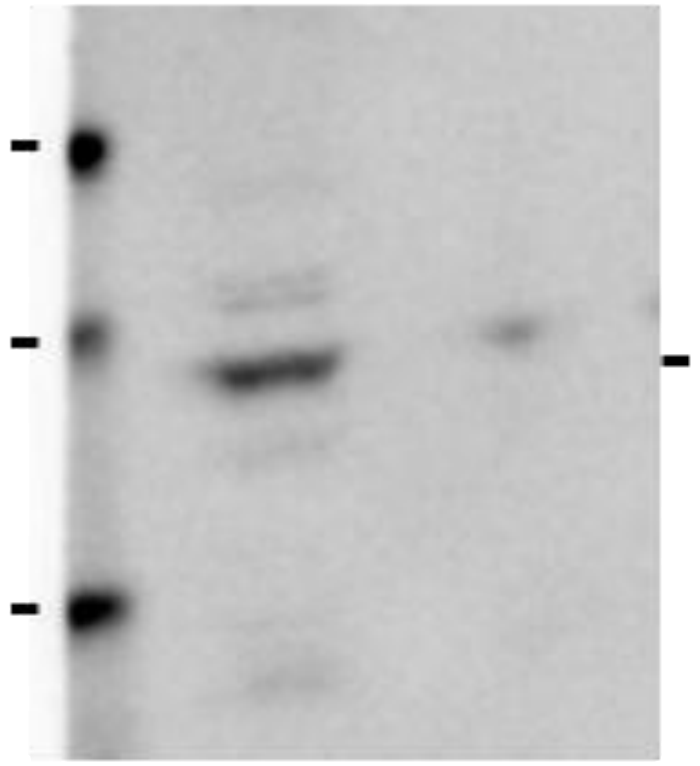

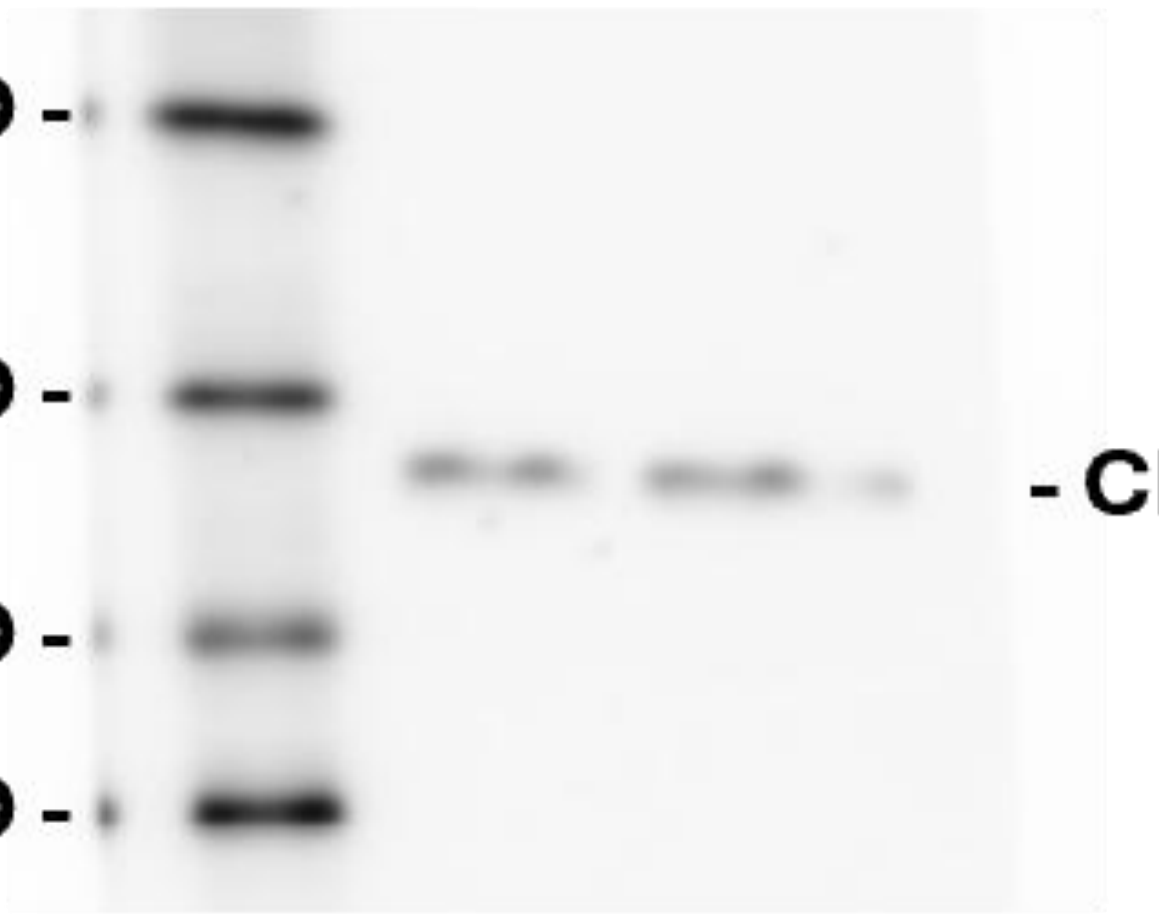

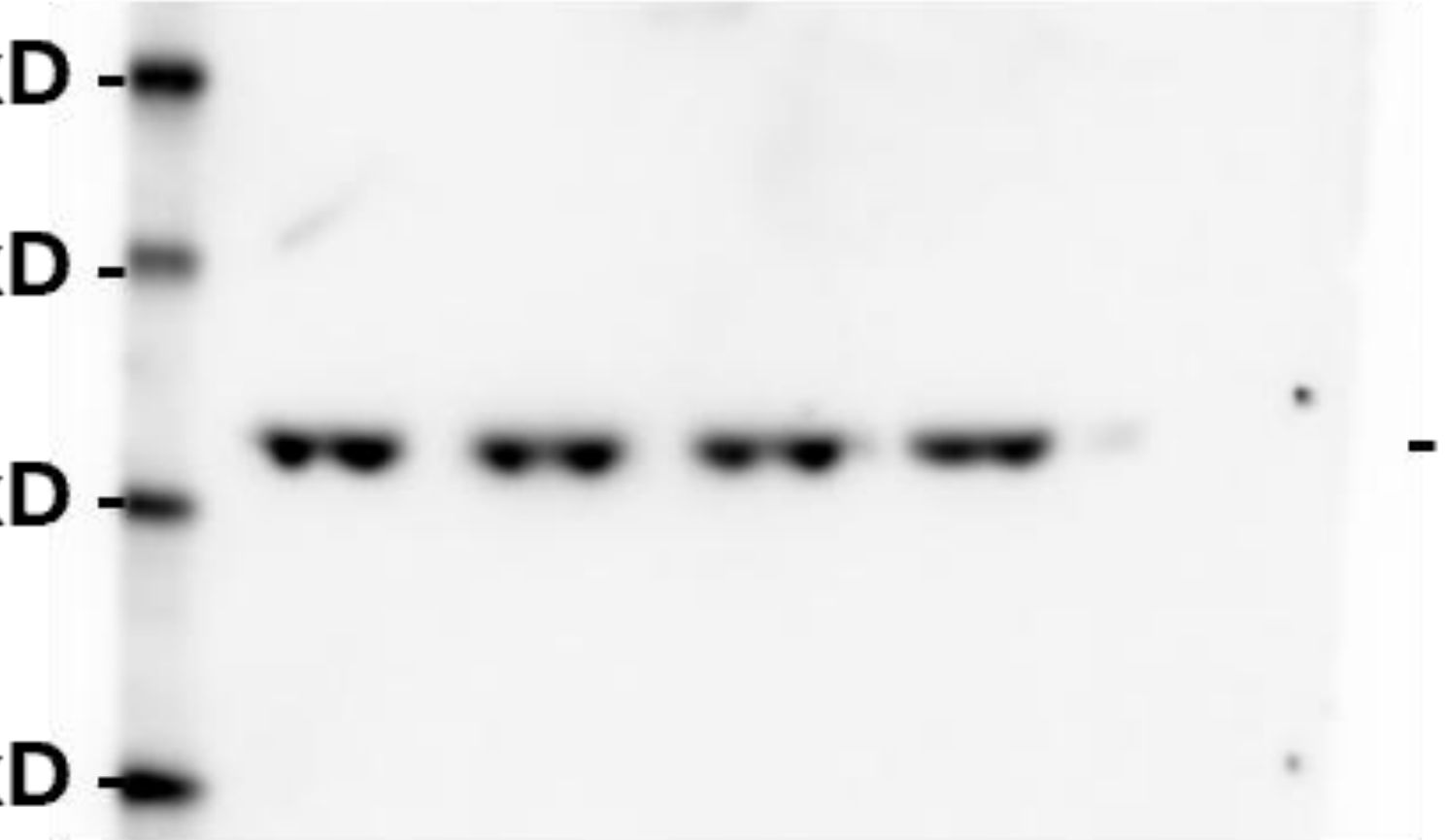

Original West Blot scans for Fig. 6E

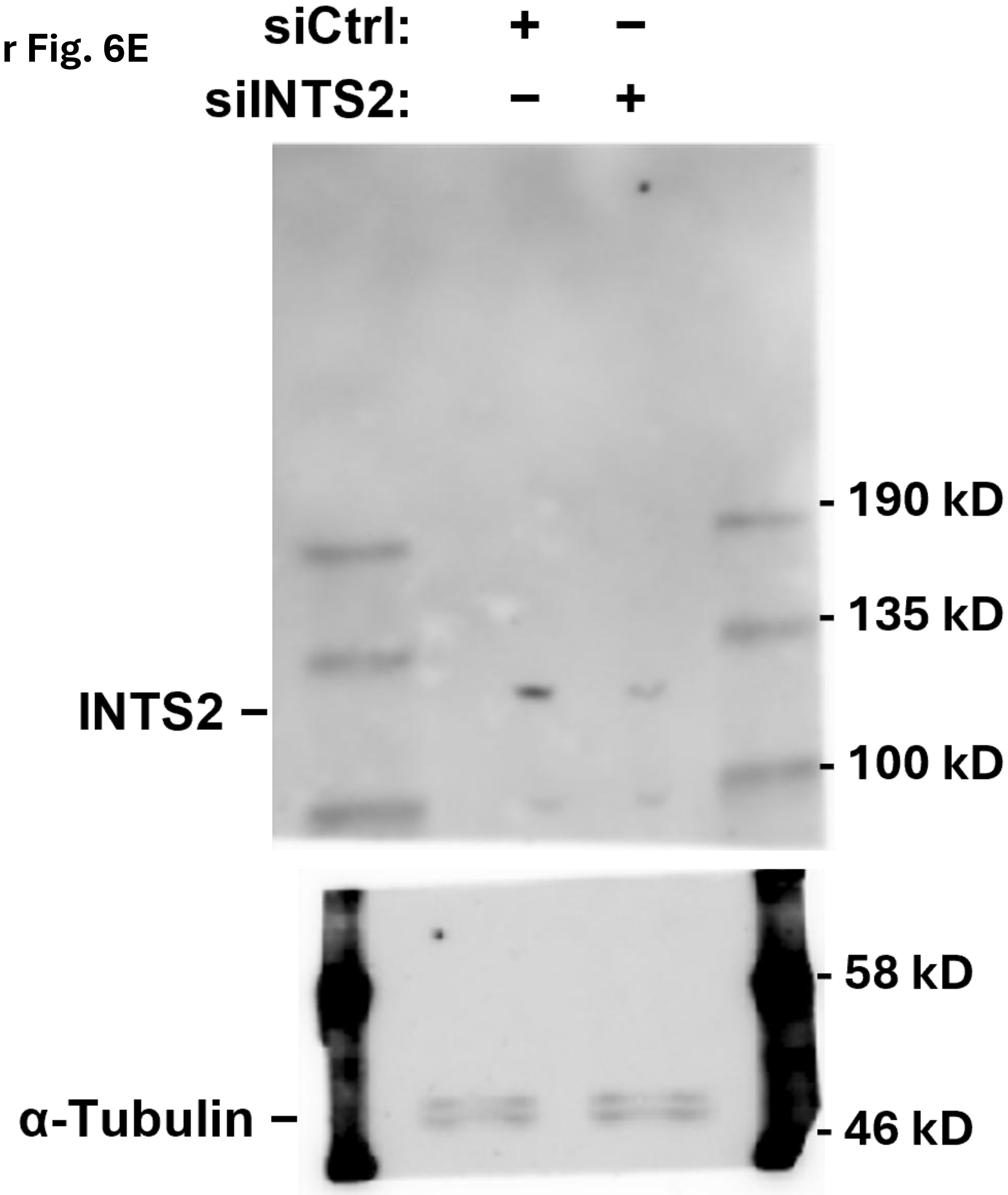

Original West Blot scans for Fig. S7B

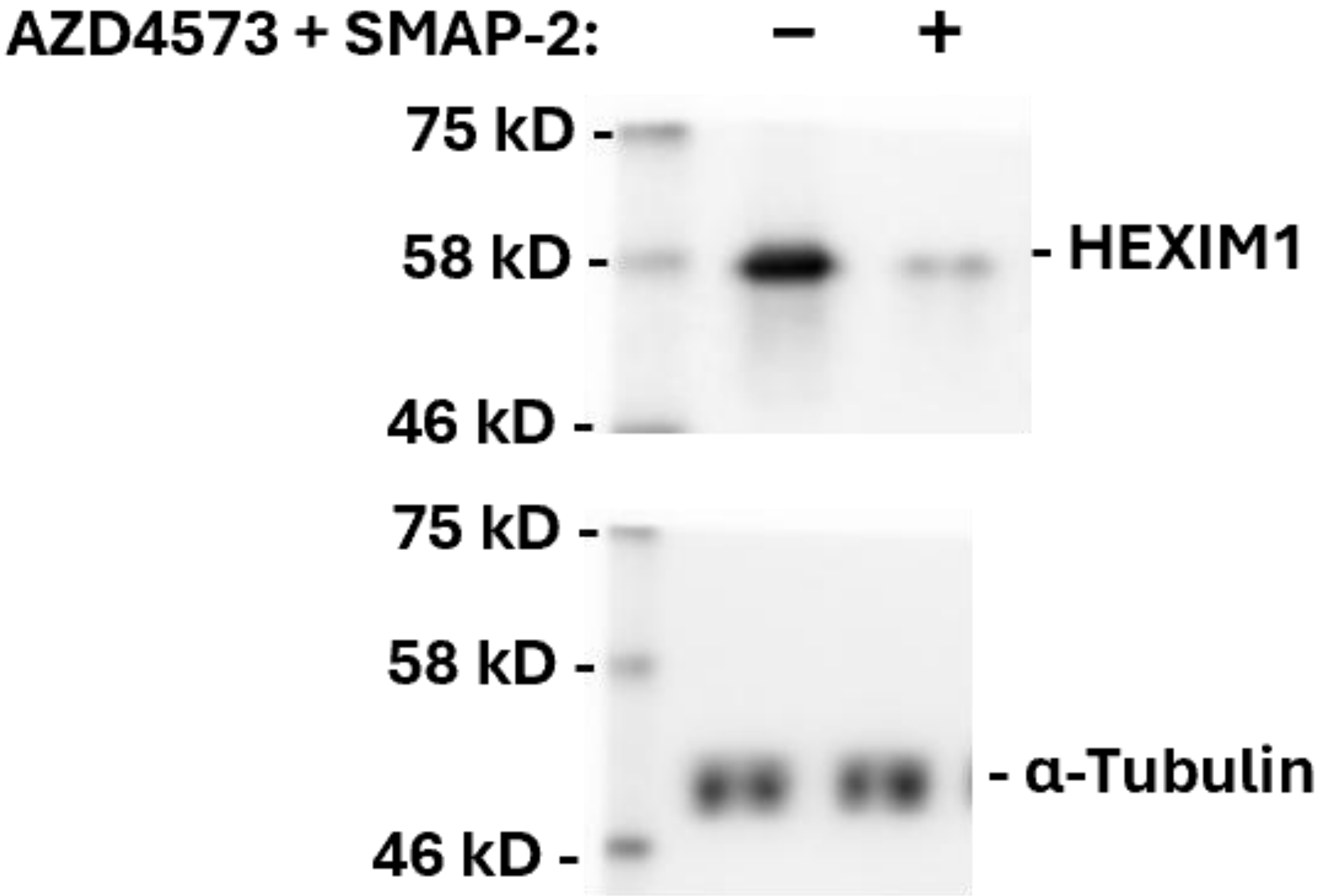

Supplement: Supplementary file 2 [file mmc4.pdf]
